# Characterization of Polyphenols from *Chenopodium botrys* after Fractionation with Different Solvents and Study of Their In Vitro Biological Activity

**DOI:** 10.3390/molecules28124816

**Published:** 2023-06-16

**Authors:** Dimitar Bojilov, Stanimir Manolov, Angelika Nacheva, Soleya Dagnon, Iliyan Ivanov

**Affiliations:** Department of Organic Chemistry, Faculty of Chemistry, University of Plovdiv, 4000 Plovdiv, Bulgaria; manolov@uni-plovdiv.net (S.M.); andjelikanacheva@gmail.com (A.N.); solbono@abv.bg (S.D.); iiiliyan@abv.bg (I.I.)

**Keywords:** *Chenopodium botrys*, in vitro biological activity, HPSA, HRSA, NOSA, IAD, ATA, flavonoid-glycosides, 6-methoxy and polymethoxylated flavones

## Abstract

In the present work, we have investigated the polyphenolic composition of *Chenopodium botrys* from Bulgaria. The polyphenols were fractionated with solvents of varying polarity (*n*-hexane, chloroform, ethyl acetate, and *n*-butanol). The fractions were analyzed by HPLC-PDA and UHPLC-MS. The ethyl acetate fraction contained mono- and di-glycosides of quercetin, di-glycosides of kaempferol, and isorhamnetin and monoglycosides of hispidulin and jaceosidine. We found quercetin triglycosides in the butanol fraction. The ethyl acetate and butanol fractions contained 168.82 mg/g Extr and 67.21 mg/g Extr of quercetin glycosides, respectively. The main components of the polyphenolic complex in *C. botrys* were 6-methoxyflavones (355.47 mg/g Extr), which were found in the chloroform fraction. The flavonoids pectolinarigenin, demethylnobiletin, and isosinensetin, and the glycosides of quercetin (triglycosides, acylglycosides), kaempferol, isorhamnetin, hispidiulin, and jaceosidine, were discovered and reported in *Chenopodium botrys* for the first time. We used in vitro methods to assess the biological activity against oxidative stress (hydrogen peroxide scavenging activity (HPSA) and hydroxyl radical scavenging activity (HRSA)), nitrosative stress (nitric oxide scavenging activity (NOSA)), anti-inflammatory activity (IAD inhibition), and anti-tryptic activity (ATA). Quercetin mono- and di-glycosides exhibited greater HPSA and HRSA (IC_50_ = 39.18, 105.03 µg/mL), while 6-methoxyflavones had a greater NOSA (IC_50_ = 146.59 µg/mL). The same components showed the highest ATA (IC_50_ ranging from 116.23 to 202.44 µg/mL).

## 1. Introduction

The genus *Chenopodium* (Family *Chenopodiaceae*) includes more than 200 species native to much of Europe, Asia, India, China and the Americas [1]. *Chenopodium botrys* is a representative of this family. Synonyms of *C. botrys* are *Ambrosia mexicana hort*, *Dysphania botrys* L., *Mosyakin* & *Clemants*, and *Ambrina botrys* (L.) Moq. [1]. In many different countries and cultures, *C. botrys* extract is used to treat various conditions and diseases. In Iranian folk medicine it is used as an expectorant and tonic, while in France and southern Europe it is used to treat asthma [2,3]. In Serbian traditional medicine, the dried aerial parts are used to prepare decoctions or liquid extracts as medicine with diuretic, antispasmodic and antidiarrheal properties, and sometimes as a spice [4]. In Pakistan, the young leaves and branches of *C. botrys* are used to heal wounds, and an infusion of the whole plant is used orally to treat stomachaches and headaches, and as a laxative and diuretic [5]. In some states in India, *C. botrys* is used as a popular flavoring for meat, cheese and barley soup. Additionally, in Indian medicine, it is used as an anthelmintic, diuretic and laxative. In the Kashmir Himalayas, a decoction of the seeds is taken in cases of tapeworm infection, especially in children [6]. It also has an analgesic effect and is used for headaches, colds and flu [1].

More is known about the health benefits of *C. botrys* than its chemical composition. The essential oil is known to be of great interest. The literature mainly reports on the composition of the essential oil, as it is known that it can be divided into two chemotypes—rich in monoterpenes [7,8,9] and rich in sesquiterpenes [10,11,12,13,14,15].

In fact, data on the content of polyphenols are quite scarce. There are only few studies on the flavonoid composition of this plant species, mostly focused on the isolation of two glycosides of the flavonol quercetin (quercetin-3-*O*-*β*-D-glucopyranoside) and quercetin-3-*O*-*β*-(D-glucopyranosyl-6-*β*-D-glucopyranoside), and flavones chrysoeriol, hispidulin, salvigenin, 5-methylsalvigenin, 7-methyleupatulin, sinensetin and jaceosidin [16,17,18]. In our previous studies, after fractionating the polyphenolic complex with solvents of different polarities (*n*-hexane, chloroform, ethyl acetate and *n*-butanol), we have studied only a part of the components. UHPLC-ESI-MS in positive mode was used to identify the polyphenols. In the chloroform fraction, 6-methoxy flavones were identified, as well as the polymethoxylated flavones sinensitin and nobiletin, while in the ethyl acetate fraction, rutin, quercetin-3-*O*-galactoside and quercetin-3-*O*-glucoside were found [19].

Apart from our work, there are no new reported results in this field so far. The biological activity of the polyphenols in *C. botrys* has not yet been studied in details, and there are no data on which part of the polyphenolic complex shows greater activity, or on which part of it is of greater interest for future research. In the current study, we set out to investigate the capacity of the polyphenolic composition of *C. botrys* in relation to the prevention of harmful radicals resulting from oxidative and nitrosative stress, as well as in vitro anti-inflammatory and anti-arthritic activity. To reach the objective, many crucial actions must be completed, such as fractionation of the polyphenol composition with solvents of different polarity. Using this procedure, polyphenols may be divided into polar, slightly polar, and non-polar polyphenol groups. The second action is the HPLC-DAD and UHPLC-MS analysis of individual groups of polyphenols. This is associated with the identification, quantification, and mass spectral analysis of polyphenol structure. The third task is an investigation of the structure–activity relationship of different groups of polyphenols.

## 2. Results and Discussion

### 2.1. Fractionation of Polyphenolic Complex with Different Solvents and Fraction Yields

Fractionation is a key step in both polyphenol analysis, particularly with UHPLC-MS/MS, and biological activity assessment. The components of the polyphenolic complex are separated by polarity using fractionation. Solvents with varying polarities were used, as follows: hexane, chloroform, ethyl acetate, and butanol.

This stage allows for the completion of the two major tasks. The first objective is connected with removing the matrix’s impact and facilitating the identification of components. The second goal is to determine which component of the polyphenolic complex is high in vitro biological activity, and to establish a link between structure and activity.

**Figure 1:**
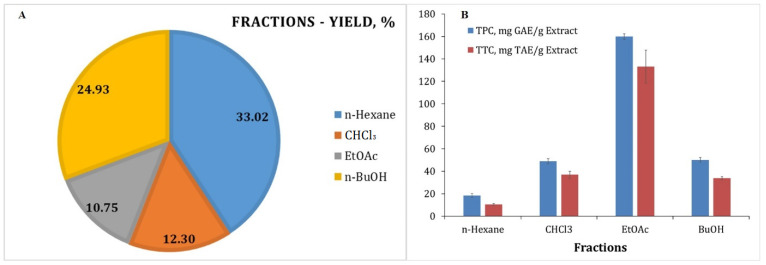
Yields (%) of the hexane, chloroform, ethyl acetate and butanol fractions. The experiments were performed in triplicate (**A**). Total phenolic content (TPC) and total tannin content (TTC) of different fractions obtained from *C. botrys*. The TPC and TTC results are presented as mg GAE/g Extr and mg TAE/g Extr, respectively (**B**).

The highest yield was obtained in the hexane fraction (33.02%), followed by the butanol fraction (24.93%), and the lowest yield was determined in the ethyl acetate fraction (10.75%) (Table 1, Figure 1A).

### 2.2. Content of Total Phenols (TPC) and Tannins (TTC) in the Fractions

Polyphenols, as secondary metabolites, are abundant in the plant world, and contribute considerably to antioxidant and other properties that may benefit human health. As a result, when researching a specific herb, it is critical to assess the total phenolic content and total tannin content of the various extracts, fractions, and tinctures.

In the three fractions, the TPC values were between 18.41 and 159.84 mg GAE/g Extr, while TTC values ranged between 10.40 and 133.08 mgTAE/g Extr. Nevertheless, the ethyl acetate fraction was the smallest, and the TPC and TTC data showed the highest yield of phenolic compounds (Table 1 and Figure 1B). Thus, the ethyl acetate fraction was found to be the richest in total phenolics (159.84 mg GAE/g Extr) and total tannins (133.08 mgTAE/g Extr). The biological activity is directly proportional to the quantity of polyphenols in the extracts.

### 2.3. Content of 6-Methoxy Flavones and Quercetin Glycosides in the Fractions

An important goal of the study was estimating the total amount of flavonoids and glycosides in the fractions that contained the polyphenolic complex from *C. botrys*. TPC is a generic indication and does not provide detailed information about the polyphenolic composition. Chromatographic analysis offers detailed information on the concentration and distribution of the herb’s different polyphenol classes. Previously, we examined the 70% methanol extract of *C. botrys* and established the fingerprint profile of the polyphenolic complex, which consists mainly of 6-MFs and a small amount of quercetin glycosides [19].

**Figure 2:**
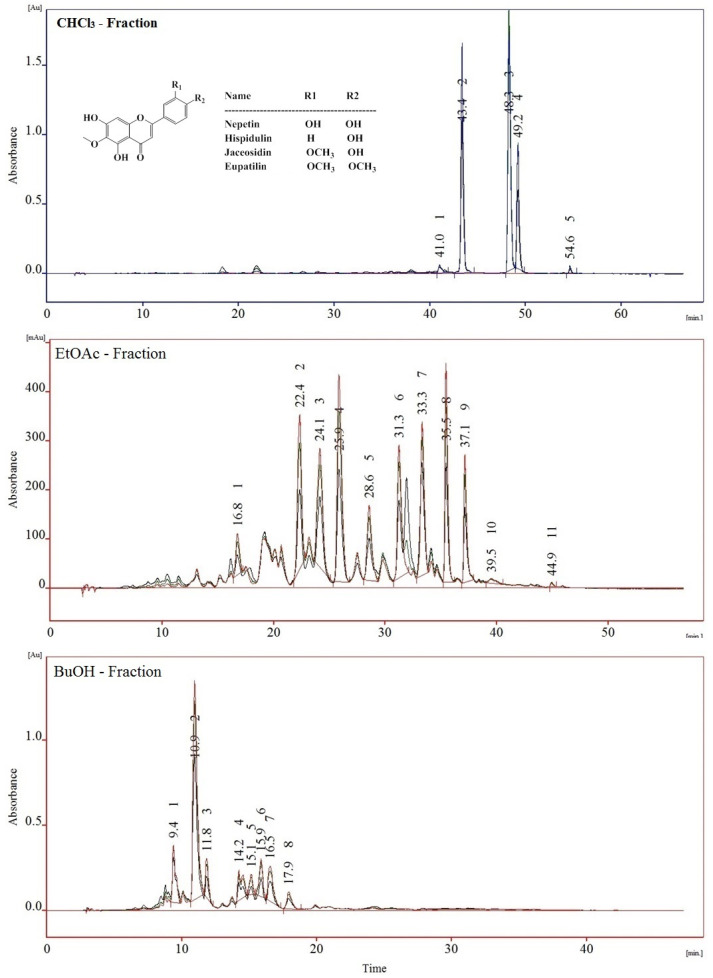
Chromatographic profiles of the fractionated polyphenolic complex in *Chenopodium botrys* obtained by HPLC-PDA. The chloroform fraction contains mainly 6-methoxy and polymethoxylated flavones, the ethyl acetate fraction contains mono- and di-glycosides of quercetin, kaempferol, isorhamnetin, monoglycosides of hispidulin, jaceosidin and isorhamnetin, and the butanol fraction contains triglycosides of quercetin.

In the present research, the polyphenolic complex was fractionated by polarity using several organic solvents, including hexane, chloroform, ethyl acetate, and butanol. The last three fractions were analyzed by HPLC-PDA. From the chromatographic profiles, we have found that quercetin glycosides were most effectively extracted with polar solvents, such as ethyl acetate and butanol, while 6-MFs were most effectively extracted with the less polar chloroform [19] (Figure 2).

Using a combination of HPLC-PDA and UHPLC-MS/MS analysis, we have discovered that ethyl acetate was able to extract mono- and di-glycosides of quercetin, kaempferol, isorhamnetin, hispidulin, and jaceosidine, while butanol was able to extract the more polar quercetin triglycosides. Quercetin glycosides are shown to be the main constituents in both fractions.

The content of quercetin glycosides and 6-methoxy flavones was determined relative to quercetin and hispidulin, respectively. The study results reveal that the ethyl acetate fraction has the highest level of quercetin glycosides (168.82 mg/g Extr), whereas the butanol fraction has a 2.5-fold lower content. The glycosides of kaempferol, isorhamnetin, hispidulin, and jaceosidine are present in low concentrations in the same fraction. The amount of 6-MFs in the chloroform fraction reached 355.47 mg/g Extr (Table 1).

### 2.4. Identification of Polyphenolic Components by UHPLC-MS/MS

#### 2.4.1. Flavonoids Identification

**Figure 3:**
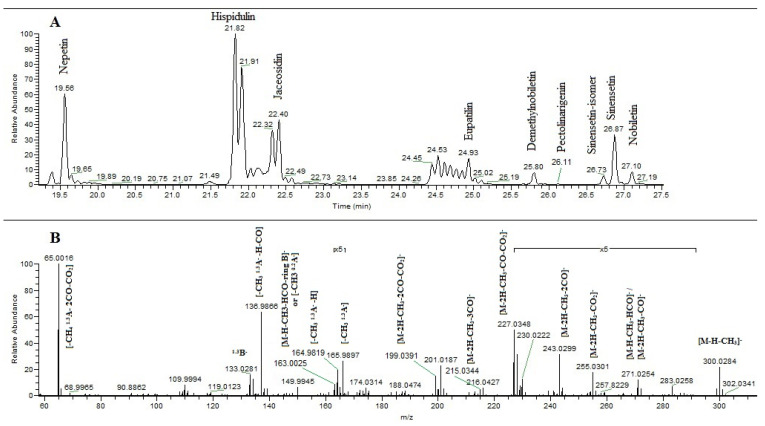
Total ion current (TIC) of flavonoids from *C. botrys* in chloroform fraction (**A**); mass spectrum of nepetin obtained by negative ion ESI-MS/MS (**B**).

In our previous studies, we used UHPLC-MS in the positive mode and reported for the first time 6-MFs as major components in *C. botrys* [19]. Until now, this group of polyphenols has gained little attention. Therefore, in the present work, we have investigated their structure under the conditions of ESI-MS/MS in the negative mode. Under these conditions, lower noise was observed. Both ionization modes are useful and have aided in the elucidation of the compounds’ complex structures. In addition, different fragmentation pathways were observed. Upon further investigation, in addition to the known compounds, we completed the group of methoxyflavones with pectolinarigenin, demethylnobiletin, and the sinensetin isomer isosinensetin, which have not yet been reported in the polyphenolic composition of *C. botrys* (Figure 3A). The fragmentation of 6-MFs showed a well-defined relationship, but the fragmentation mechanism of polymethoxylated flavones was reliant on the quantity of methoxy groups.

**Scheme 1:**
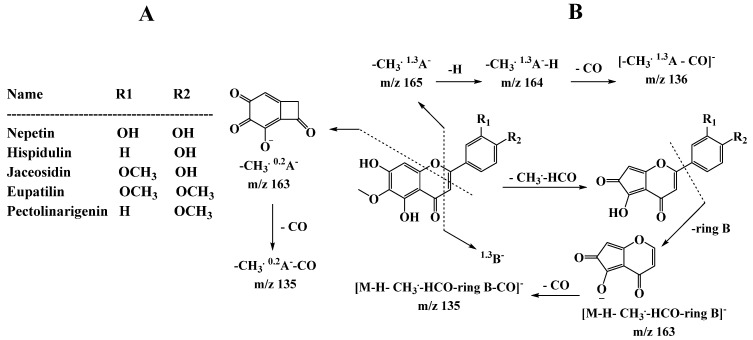
Structures of 6-methoxyflavones (**A**); nomenclature for the different retro-cyclization cleavages in negative ESI-MS/MS mode (**B**).

On Scheme 1, labels ^i,j^A and ^i,j^B refer to the fragments containing A^−^ and B^−^rings, respectively, and the superscripts i and j indicate the C ring bonds that have been broken. The ^i,j^A^−^ and ^i,j^B^−^ions undergo further fragmentation by losing a methyl group (–CH_3_), a –HCO group, or small neutral molecules, such as CO and CO_2_.

**Scheme 2:**
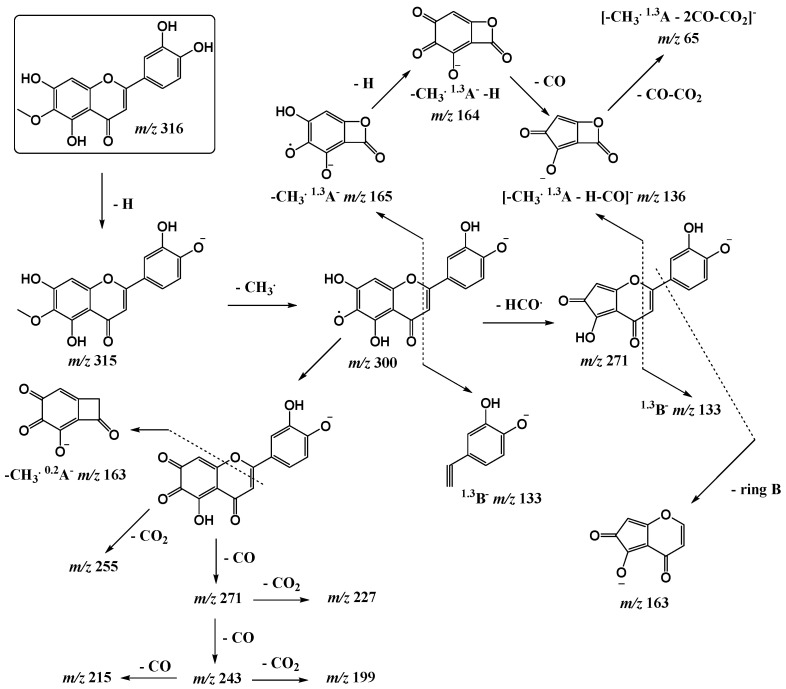
Proposed fragmentation of deprotonated nepetin [M−H]^−^ at CE 55 eV.

The mass spectrum of 6-methoxyflavone nepetin obtained from [M-H]^−^ by negative ion fragmentation in ESI-MS/MS mode is presented on Scheme 2. The fragmentation pathways of nepetin are shown on Scheme 2. The same ion cleavages were observed for the other 6-MFs-hispidulin, jaceosidin, eupatilin and pectolinarigenin. Initially, the 6-MFs molecular ion loses a methyl radical to give the ion [M–H–CH_3_]^−^. This ion loses a radical –CHO by producing [M–H–CH_3_–CHO]^−^ or [M–2H–CH_3_–CO]^−^ ions (Scheme 1 and Scheme 2). The resulting ion [M–H–CH_3_–CHO]^−^proves the presence of methoxy group at the sixth position in ring A. Furthermore, the same ion systematically undergoes the loss of small neutral molecules, such as CO and CO_2,_ leading successively to the formation of ion *m*/*z* 243, ion *m*/*z* 215 and ion *m*/*z* 227 (Scheme 2). The loss of neutral molecules CO and CO_2_ may be attributed to ring C. Similarly, it undergoes the fragmentation of hispidulin, jaceosidin, eupatilin and pectolinarigenin (Table 2).

The fragmentation of nepetin involves the cleavage of C–C bonds at position 1/3 and 0/2 of the C ring, and gives structurally informative ^1,3^A^−^, ^1,3^B^−^ and ^0,2^A^−^ ions (Scheme 2). The intensity of the fragment ions depends on the collisional energy (CE). RDA fragmentation may occur in nepetin, producing *m*/*z* 164 and *m*/*z* 133 ions (Scheme 2, Figure 3B). In the high CE spectrum of deprotonated nepetin (Scheme 2, Figure 3B), the ion *m*/*z* 164 corresponds to the fragment [–H–CH_3_-^1,3^A^−^/–CH_4_-^1,3^A^−^], which is characterizing by a direct loss of methyl group, and it is not an intensive peak. Ion *m*/*z* 133 shows low intensity corresponding to the fragment ^1,3^B^−^. Furthermore, ion [–H–CH_3_-^1,3^A^−^/–CH_4_-^1,3^A^−^] systematically undergoes the loss of CO, leading to the more intensive ion with *m*/*z* 136 [–CH_4_-^1,3^A^−^–CO] (Scheme 2, Figure 3B). Ion *m*/*z* 136 additionally loses CO and CO_2_, resulting in the formation of the more intense ion with *m*/*z* 65 [–CH_4_-^1,3^A^−^–2CO–CO_2_]. The intensity of ion *m*/*z* 136 depends on the presence of polar groups in ring B. The presence of OH groups leads to an increase in its intensity. The *m*/*z* 163 ion was obtained by two pathways. One is the retrocyclization of ring C (*0*/*2*), and the resulting ion is –CH_3_ ^0.2^A^−^, and the second pathway involves the cleavage of the C2–C1′ bond between rings C and B, yielding an ion [M–H–CH_3–_HCO–ring B]^−^. Which one is the most likely to be seen is difficult to determine, since the elemental composition of the two ions is the same, i.e., isomeric ions (Scheme 2, Figure 3B).

The intensity of ion *m*/*z* 163 depends on the functional groups (OH or OCH_3_) in ring B. Therefore, in nepetin and hispidulin, ion *m*/*z* 163 is of low intensity due to the presence of OH groups. The presence of OCH_3_ groups increases the intensity of ion *m*/*z* 163. The same ion in jaceosidine, eupatilin and pectolinarigenin loses a neutral molecule CO, and an ion *m*/*z* 135 [–CH_3_ ^0.2^A^−^–CO] or [M–H–CH_3_–HCO–ring B–CO]^−^ is obtained. The ions *m*/*z* 164, *m*/*z* 163, *m*/*z* 136 and *m*/*z* 65 are specific to the fragmentation of nepetin in negative mode. They contribute to the identification of 6-MFs being characteristic for the substitution in the A ring. For 6-MFs, these ions characterize the substituents in the A ring. Significant differences in the fragmentation of 6-MFs appear only in the substitution in the B ring. The mass spectra of [M − H]^−^ ions of 6-MFs are summarized in Table 2 and Supplementary Material Figures S1–S8.

In addition to 6-MFs, with the capabilities of UHPLC-MS/MS, we have identified the polymethoxylated flavones (PMFs) demethylnobiletin (t_R_ = 25.80) and an isomer of sinensetin (t_R_ = 26.73) (Figure 3A). Previously, we reported the presence of sinensetin and nobiletin [19]. The loss of CH_3_ groups in PMFs under ESI-MS/MS negative mode results in stable quinoid forms (Figures S9–S15). The same forms undergo rearrangement with the release of neutral molecules CO and CO_2_. The resulting fragment ions correspond to [M–H–*i*CH_3_–*i*CO]^−^ and [M–H–*i*CH_3_–*i*CO–CO_2_]^−^ (Figures S10, S14 and S15).

Sinensetin and its isomer have similar spectra, which can be seen in both ionization modes (Figures S9 and S11). The mass spectra of both isomers show that the fragmentation mechanism approaches that of the 6-MFs. The reason for this is that ring A contains three substituents, and under the conditions of ESI-MS/MS (negative and positive mode), after the cleavage of CH_3_ groups, ring A regroups in a stable quinoid form (Figures S10 and S12). In order to more thoroughly examination of the structure of the two isomers, we have used two ionization regimes. The cleavage of CH_3_ groups and the subsequent RDA cyclization (1/3) of ring C leads to the formation of ion *m*/*z* 168 ([–3CH_3_ ^1.3^A^+^]), which systematically loses neutral molecules CO and leads to the receiving of the ions *m*/*z* 140 ([–3CH_3_ ^1.3^A^+^–CO]) and *m*/*z* 112 ([–3CH_3_ ^1.3^A^+^–2CO]) (Figure S12).

After ion 168 underwent retrocyclization, the product ion 99 was produced (Figure S12). The second product ion from RDA cyclization has *m*/*z* 147 ([–CH_3_ ^1.3^B^+^]), which also loses CO, and the product fragment (*m*/*z* 119) corresponds to ([–CH_3_ ^1.3^B^+^–CO]) (Figure S12). In the negative mode, the same RDA cyclization is also seen (Figure S10). In this instance, the resultant fragments differ structurally. Important details about the two isomers’ structures are revealed by both modes. We suggest that the isomer is most likely isosinensetin.

The fragmentation of PMFs in negative mode is triggered by an increase in methoxy groups, and is carried out by the mechanisms of nobiletin and demethylnobiletin. In this instance, characteristically, only methyl groups are cleaved in order to produce a stable quinoid form. The majority of the molecules eliminated are neutral molecules, such as CO and CO_2_. RDA cyclization yields the ions *m*/*z* 132 and *m*/*z* 133 (Figures S13–S15).

As a consequence of this research, we can infer that in *C. botrys*, three novel compounds (pectolinarigenin, sinensetin isomer, and demethylnobiletin) were identified that have not been reported before now.

#### 2.4.2. Identification of Glycosides

*C. botrys* is the least researched member of the *Chenopodiaceae family*, particularly because of its polyphenol content. Metabolism in various species of *Chenopodiaceae* is known to synthesize mainly glycosides of quercetin, kaempferol and isorhamnetin. The carbohydrates that are involved in the structure of glycosides are glucose, galactose, rhamnose and apiose [18,20,21,22,23,24,25,26,27,28,29,30,31,32,33,34,35,36].

**Figure 4:**
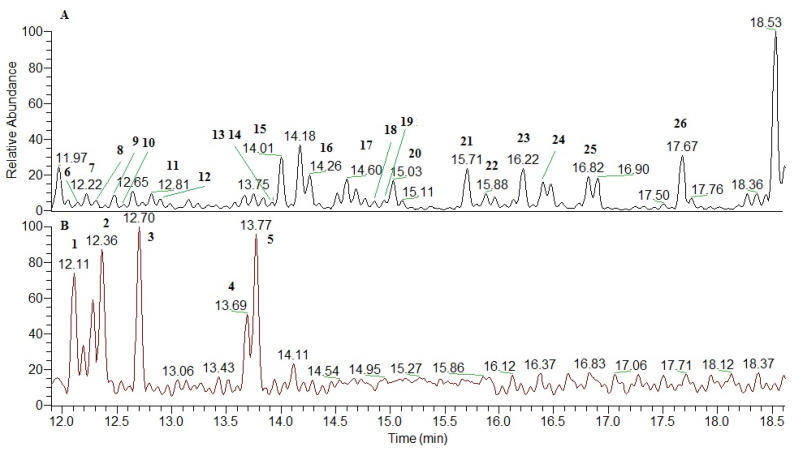
Total ion current (TIC) of flavonoids from *C. botrys* in EtOAc fraction (**A**); total ion current (TIC) of flavonoids from *C. botrys* in BuOH fraction (**B**). Mono- and di-glycosides of quercetin (Peaks—**6**–**9**, **11**, **12**, **15**, **20**, **21**, **23**–**26**) were identified in the EtOAc fraction (**A**), di-glycosides of kaempferol (Peaks—**18**, **22**), mono- and di-glycosides of isorhamnetin (Peaks—**10**, **13**, **16**), monoglycosides of jaceosidine (Peaks—**14**, **19**) and hispidulin (Peak—**17**). Triglycosides of quercetin were identified in the BuOH fraction (**B**) (Peaks—**1**–**5**).

*C. botrys* metabolism is quite diversified, since the main components are 6-MFs, and the glycosides are mostly quercetin, followed by kaempferol, isorhamnetin, and in tiny amounts jaceosidine and hispidulin (Figure 4, Table 3). The glycosides were investigated using ESI-MS/MS in negative mode. From the mass spectra, we have found that the fragmentation of the molecular ion [M–H]^−^ produces an ion (*Y_o_*) due to the loss of carbohydrate units. The fragmentation data were compared to the fragmentation of the standards rutin (peak **6**), quercetin 3-*O*-galactopyranoside (peak **11**) and quercetin 3-*O*-glucopyranoside (peak **12**) (Table 3). In the ESI-MS spectrum, the molecular ion of rutin [M–H]^−^ with *m*/*z* 609 was observed (Figure S16). Its fragmentation produces an ion with *m*/*z* 301(*Y_o_*) ([M–H–146–162]^−^) that is related with the loss of the disaccharide rutinose (308 Da). In addition, we detected an ion with *m*/*z* 463, which, following fragmentation, produces an ion with *m*/*z* 301, but one carbohydrate unit of 162 Da (glucose, galactose) is lost. The ESI-MS/MS experiment and fragmentation of ion 301 showed the formation of ions with *m*/*z* 179 [^1.2^A^−^], *m*/*z* 151 [^1.2^A^−^–CO] and *m*/*z* 107 [^1.2^A^−^–CO–CO_2_], which are characteristic of the aglycone quercetin (Figure S16, Table 3). This method simplifies the identification and fragmentation of the glycosides found in the two polar fractions.

Peak **1** with t_R_ = 12.11 min (Figure 4B, Table 3) was identified as quercetin 3-*O*-(2″-*O*-apiofuranosyl-6″-*O*-glucopyranosyl)-*β*-D-glucopyranoside. In the MS/MS experiment, the molecular ion *m*/*z* 757 lost a pentose unit of 132 Da, and an ion *m*/*z* 625 ([M–H–132]^−^) was obtained. The same ion lost 162 Da, and the resulting ion corresponds to [M-H-132-162]^−^ with *m*/*z* 463, which lost another 162 Da, and the resulting ion 301 fragmented into the characteristic quercetin ions *m*/*z* 179 [^1.2^A^−^], *m*/*z* 151 [^1.2^A^−^–CO] and *m*/*z* 107 [^1.2^A^−^–CO–CO_2_]. The derivatives of the aglycone quercetin were discovered to be peaks **2**–**4** (Figure 4B, Table 3). All three glycosides are isomers with the molecular ion *m*/*z* 841. According to mass spectrum analyses, the molecular ion’s structure comprises two units of rhamnose and one hexose (glucose and galactose). Furthermore, the glycoside is acylated with malonic acid. During the MS/MS experiment, the molecular ion sequentially lost two units of rhamnose (2 × 146 Da) and malonic acid, yielding a *m*/*z* 463 ion. The resulting ion from the MS/MS experiment was quercetin, and its specific fragment ions were *m*/*z* 179 [^1.2^A^−^], *m*/*z* 151 [^1.2^A^−^–CO] and *m*/*z* 107 [^1.2^A^−^–CO–CO_2_]. Peak **5** with t_R_ 13.77 min (Figure 4B, Table 3) is molecular ion *m*/*z* 883, and was identified as Quercetin 3-*O*-*α*-L-rhamnopyranosyl-*α*-L-rhamnopyranosyl-*β*-D-malonylglucopyranoside (acyl). The molecular ion lost an acyl group, yielding the ion *m*/*z* 841 ([M–H–acyl]^−^). This indicates that the molecular ion was acylated with acetic and malonic acids. The same ion was structurally identical to the one before it, and fragments in the same way.

Based on the examination of quercetin glycosides in the ethyl acetate fraction and MS/MS data, we discovered that the fragmentation of the di-glycosides was identical to that of rutin (Table 3). We discovered mono- and di-acyl glycosides of quercetin (peaks **8**, **9**, **15**) in the polyphenol composition. Peak **8** (t_R_ 12.31 min) was identified as Quercetin-*O*-(acyl)-hexoside-hexoside with molecular ion *m*/*z* 667 (Figure 4A, Table 3).

It lost an acyl group and produced the ion *m*/*z* 625 ([M–H–acyl]^−^), which is an ion with two hexoses in its structure. The resulting ion gradually lost 162 Da, generating the ions *m*/*z* 463 ([M–H–acyl–162]^−^) and *m*/*z* 301 ([M–H–acyl–162–162]^−^). Fragment ions *m*/*z* 179 [^1.2^A^−^], *m*/*z* 151 [^1.2^A^−^–CO] and *m*/*z* 107 [^1.2^A^−^–CO–CO_2_] prove that the molecular ion *m*/*z* 667 is a quercetin glycoside. Peaks **9** and **15** are distinguished by the same molecular ion, *m*/*z* 709. We identified them as quercetin diacyl glycosides using MS/MS data. The molecular ion lost an acyl group, yielding an ion *m*/*z* 667 ([M–H–acyl]^−^), which then lost a neutral molecule CO, yielding an ion *m*/*z* 639 ([M–H–acyl–CO]^−^). Furthermore, the molecular ion lost 204 Da (acyl+hexose) and produced ion *m*/*z* 505 ([M–H–acyl–hexose]^−^). The same ion lost another acyl group, and the resulting ion was *m*/*z* 463 ([M–H–2acyl–hexose]^−^). The resulting ion from the MS/MS experiment was quercetin, and its characteristic ions were *m*/*z* 179 [^1.2^A^−^], *m*/*z* 151 [^1.2^A^−^–CO] and *m*/*z* 107 [^1.2^A^−^–CO–CO_2_].

Peak **18** (t_R_ 14.85 min) (Figure 4A, Table 3) was identified as Kaempferol-*O*-(acyl)-hexoside-*O*-(acyl)-hexoside with molecular ion *m*/*z* 693. It lost 204 Da immediately, yielding the *m*/*z* 489 ion, and then lost an acyl group, yielding the *m*/*z* 447 ion. The same ion lost 162 Da and produced an ion with *m*/*z* 285. Under ESI-MS/MS conditions, it fragmented to the ions *m*/*z* 151 [^1.3^A^−^], *m*/*z* 125 [^1.4^A^−^] and *m*/*z* 107 [^1.3^A^−^-CO_2_].

Peaks **10**, **13** and **16** (Figure 4A and Figure S17B, Table 3) were identified as isorhamnetin glycosides. Peaks **10** and **13** have the molecular ion *m*/*z* 623, indicating a di-glycoside comprising glucose and rhamnose. They were identified as Isorhamnetin 3-*O*-glucopyranoside-7-*O*-rhamnopyranoside and Isorhamnetin 3-*O*-glucopyranoside-rhamnopyranoside. The glycoside **10** (Peak **10**) lost 162 Da and produced ion *m*/*z* 461 [M–H–162]^−^. The same ion lost rhamnose moiety, and the ion 315 [M–H–162–146]^−^ corresponds to isorhamnetin. Glycoside **13** (Peak **13**) successively lost 146 Da; the product ion 477 corresponded to [M–H–146]^−^, which then lost another 146 Da. The resulting ion [M–H–146–162]^−^ was isorhamnetin. The characteristic ions for isorhamnetin are *m*/*z* 151[^1.3^A^−^], *m*/*z* 137 [–CH_3_ ^0.2^B]^−^, *m*/*z* 125 [^1.4^A^−^] and *m*/*z* 107 [^1.3^A^−^–CO_2_].

Peak **16** was identified as Isorhamnetin 3-*O*-glucopyranoside with molecular ion *m*/*z* 477. The fragmented ion was 315. The characteristic ions for the same flavonol are *m*/*z* 151[^1.3^A^−^], *m*/*z* 137 [–CH_3_ ^0.2^B]^−^, *m*/*z* 125 [^1.4^A^−^] and *m*/*z* 107 [^1.3^A^−^–CO_2_].

Peaks **14** and **19** (Figure 4A and Figure S17C, Table 3) were identified as Jaceosidin 7-*O*-glucopyranosid and Jaceosidin 4′-*O*-glucopyranosid. These are isomers with molecular ion *m*/*z* 491. During the fragmentation, it produces stable quinoid ion 328. The specific ions that characterize the aglycon jaceosidin are *m*/*z* 164 [CH_3_^1.3^A^−^–H], *m*/*z* 163 [CH_3_^0.2^A^−^], *m*/*z* 147 [^1.3^B^−^], *m*/*z* 136 [CH_3_^1.3^A^−^–CO] and *m*/*z* 133 [–CH_3_ ^1.3^B^−^].

Peak **17** (Figure 4A and Figure S17D, Table 3) was identified as Hispidulin 7-*O*-glucopyranoside with molecular ion *m*/*z* 461. An ion (*m*/*z* 297) with a stable quinoid form was produced during ESI-MS/MS. Hispidulin as 6-MF generated the characteristic ions *m*/*z* 164 [CH_3_^1.3^A^−^–H], *m*/*z* 163 [CH_3_^0.2^A^−^], *m*/*z* 136 [CH_3_^1.3^A^−^–CO] and *m*/*z* 117 [^1.3^B^−^].

### 2.5. Hydrogen Peroxide Scavenging Activity

The extremely important Cu^2+^ and Fe^2+^ ions take part as cofactors in a variety of enzymes and physiological processes in the human body. However, when they are present in their free state, they have a harmful impact, because Cu^2+^ ions accelerate the oxidation of ascorbic acid, which produces ROS such superoxide radicals (O_2_^•−^) and H_2_O_2_. Additionally, the Fenton reaction causes the Cu^2+^ and Fe^2+^ ions to interact with H_2_O_2_ and produce hydroxyl radicals (^•^OH) [37]. Proteins, DNA, and essential biological components like phospholipids are all damaged by ROS. This chemical damage is known as oxidative stress, and it is thought to be the cause of many health problems, including cancer, cardiovascular disease, atherosclerosis, and Alzheimer’s disease [38].

In addition to the oxidative stress mechanisms, the inflammatory process promotes and increases the generation of *ROS*. The most common cause of inflammation is the generation of superoxide anion radicals, which is linked to the creation of other ROS species such as H_2_O_2_. It also participates in reduction decomposition processes (known as the Haber–Weiss reaction) and organic hydroperoxides’ ROOH [39].

As a result, the study of substances with high scavenging ability for oxygen free radicals is a relevant and essential topic of research.

**Figure 5:**
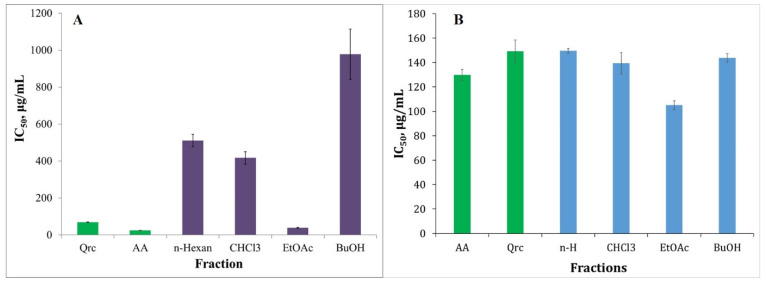
HPSA (**A**) and HRSA (**B**) of the fractionated polyphenolic complex with differences in polarity solvents obtained from *C. botrys*. Ascorbic acid (AA) and quercetin (Qrc) were used as standards. The antioxidant activity results are presented as IC_50_ (µg/mL).

The antioxidant activity of the various fractions was compared to natural substances with recognized antioxidant characteristics, such as ascorbic acid and quercetin. The antioxidant activity values of the obtained fractions ranged from 39.18 µg/mL to 978.07 µg/mL (Table 4, Figure 5A).

Except for the ethyl acetate fraction, the produced fractions had low HPSA compared to ascorbic acid (24.84 g/mL) and quercetin (69.25 g/mL). The ethyl acetate fraction had much stronger antioxidant activity than the other fractions, and was almost twice as active as the quercetin standard (Table 4, Figure 5A). The presence of glycosyl residues and methoxy groups in flavonoids influences their antioxidant action [40]. As a result, quercetin mono- and di-glycosides have a greater HPSA than methoxy flavones and quercetin triglycosides. Methoxy flavones have a low activity level. The presence of and increase in the amount of methoxy groups in ring B reduces the degree of hydrogen peroxide inhibition. However, they are more active than quercetin triglycosides. The antioxidant activity diminishes as the bulk of the glycosyl residue at the C-3 position rises [41]. This explains why the butanol fraction has a low HPSA. From the analysis, we can find that antioxidant activity is very well associated with the contents of TPC, TTC and quercetin glycosides. The *HPSA* of the fractions decreases in the following order: EtOAc > CHCl_3_ > *n*-Hexan > BuOH.

### 2.6. Hydroxyl Radical Scavenging Activity (HRSA)

The most potent oxidant is the hydroxyl radical (^•^OH), which is produced by ROS. It is produced by one-electron reductions in molecular oxygen (O_2_) in cellular metabolism, and is the primary cause of cytotoxicity in aerobic species, including humans [42].

The structure of polyphenols influences *HRSA* levels. Our standards are structurally distinct. The polyphenolic content of the obtained fractions has a flavonoid core structure. For this reason, as a reference, we utilized quercetin. The concentration gradient of quercetin glycosides is directly proportional to the antioxidant activity. Quercetin mono- and di-glycosides present in the ethyl acetate fraction showed significantly higher *HRSA* (IC_50_ = 105.03 µg/mL) (Table 4, Figure 5B), while the other three fractions showed *HRSA* that was statistically close to quercetin. From the analysis, we have established that the polar and non-polar polyphenols of *C. botrys* have a higher propensity to scavenge the highly active OH radicals (Table 4, Figure 5B). This is explained by the presence of free OH groups (C-3′ and C-4′) in ring B.

6-Methoxy flavones differ in the functional groups in ring B. They contain OH groups (hispidulin and nepetin) and OCH_3_ groups (C-3′ for jaceosidine and C-3′, C-4′ for eupatilin). The *O*-methylation of OH groups in ring B leads to a decrease in *HRSA* [40,43]. Quercetin triglycosides showed low *HRSA* compared to quercetin mono- and di-glycosides. This is due to the fact that the *O*-glycolation of the C-3 OH group and the increase in carbohydrate units leads to a decrease in antioxidant capacity [40,41]. The mass spectrum analysis confirms this result, demonstrating that the C-3 OH group is conjugated with carbohydrate units. This fact is most likely due to the low HPSA and HRSA of the butanol fraction.

### 2.7. Nitric Oxide Scavenging Activity (NOSA)

**Figure 6:**
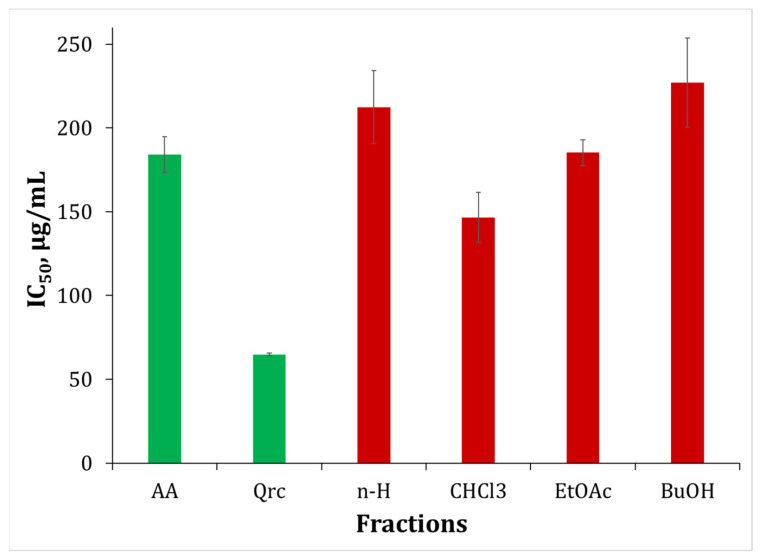
NOSA of the fractionated polyphenolic complex with different polar solvents obtained from *C. botrys*. Ascorbic acid (AA) and quercetin (Qrc) were used as standards. Antioxidant activity results are presented as IC_50_ (µg/mL).

NO is produced from the terminal guanido nitrogen atom of *L*-arginine by NO synthases (NOS), which are NADPH-dependent enzymes. NO is a bioregulatory molecule that is necessary for a variety of physiological activities, such as brain signal transmission, immunological response, vasodilation, blood pressure control, etc. By interacting with the NO molecule, the superoxide ion (O_2_^−^) generates intermediate products such as NO_2_, N_2_O_4_ and N_3_O_4_, and stable compounds such as nitrate and nitrite. It is the formation of reactive nitrogen species (RNS) known as nitrosative stress that is responsible for several pathological conditions, including cancer [44,45]. In comparison to quercetin (IC_50_ 64.82 µg/mL), the fractions had a lower NOSA. This demonstrates that the flavonoid core is more effective in quenching NO radicals. The IC_50_ values for the different fractions varied from 146.59 to 227.12 µg/mL (Table 4, Figure 6).

Similar reliance is proven for both HPSA and HRSA, i.e., quercetin mono- and di-glycosides are more active. We can deduce from this that they have the power to counteract ROS. An inverse association was seen in the deactivation of NO radicals, showing that 6-methoxyflavones displayed better NOSA than quercetin glycosides (Figure 6). Aglycones were discovered to have a higher affinity for NO radicals, and the presence of OCH_3_ groups in ring B led to the increased antioxidant activity [46]. This might explain why the chloroform fraction has a high NOSA.

### 2.8. Inhibition of Albumin Denaturation (IAD)

Inflammation is the process by which living tissues respond to stimuli caused by inflammatory factors such as physical damage, heat, microbial infections, and harsh chemical irritants. The response of the cells to inflammation will lead to certain pathological manifestations characterized by redness, heat, swelling, and pain, even causing impairments in physiological functions. Numerous disorders, including arthritis, stroke, and cancer, include inflammation as a pathogenic factor. Protein denaturation is closely linked to the initiation of the inflammatory response, which results in a variety of inflammatory disorders, including arthritis [47]. According to Opie [48], tissue injury during life might be related to the denaturation of the protein constituents of cells or of intercellular substances. Hence, the ability of a substance to inhibit the denaturation of protein represents its potential for anti-inflammatory activity.

**Figure 7:**
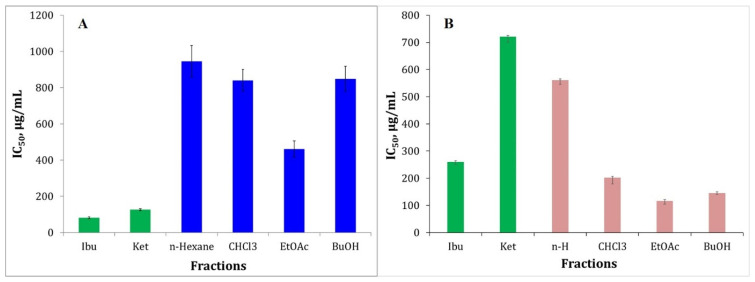
IAD (**A**) and ATA (**B**) of the fractionated polyphenolic complex with differences in polarity solvents obtained from *C. botrys*. As controls, ibuprofen (Ibu) and ketoprofen (Ket) were utilized. The results are reported as IC_50_ (µg/mL).

The resultant *C. botrys* fractions were evaluated for albumin denaturation inhibition. This approach determines the extent to which albumin may be preserved against denaturation by heating. Human albumin was employed for this purpose. Figure 7 depicts the inhibition percentages of the various *C. botrys* fractions. The study’s results are shown as IC_50_ values. We opted to utilize ibuprofen and ketoprofen as standards to examine the performance of different *C. botrys* fractions, since they have documented anti-inflammatory capabilities. The IC_50_ values of ibuprofen and ketoprofen estimated as IAD were 81.50 μg/mL and 126.58 μg/mL, respectively (Table 4, Figure 7A). The obtained data reveal that the IC_50_ values of the various fractions ranged from 461.41 to 945.06 μg/mL (Table 4, Figure 7A).

The fractions show a lower degree of protection of albumin from denaturation compared to the standards. When evaluating the degree of protection of albumin in conformity with the polarity of the fractions, the ethyl acetate fraction showed the highest activity. This suggests that the configuration of polar quercetin mono- and di-glycosides has a higher propensity for allosteric binding to the albumin molecule. In this way, it is stabilized and resistant, and prevents the denaturation of albumin. As a result, the ethyl acetate fraction is ideal for extracting physiologically active quercetin mono- and di-glycosides. The activity of quercetin triglycosides did not differ significantly from that of 6-methoxy flavones.

### 2.9. Antitryptic Activity (ATA)

Proteinases have been identified as the root cause of arthritic disorders. Neutrophils are known to be a significant source of proteinases, with a substantial number of serine proteinases in their lysosomal granules. Proteinase, which is found in leukocytes, is known to play a major role in tissue damage during inflammatory responses, and proteinase inhibitors give considerable protection [49].

In vitro anti-arthritic activity was assessed as anti-tryptic activity [49]. The purpose of this study is related to the inhibition of the enzyme trypsin, which is also from the group of serine proteinases.

The resultant *C. botrys* fractions were tested for *ATA* (Table 4, Figure 7B). The *IC_50_* values for *ATA* varied from 116.23 to 561 µg/mL. The results of the experiment reveal that the polar fractions had a stronger inhibitory efficacy than ibuprofen and ketoprofen. Higher *ATA* (116.23–202.44 µg/mL) was observed in ethyl acetate, butanol, and chloroform fractions (Figure 7B). As a result of the investigation, we discovered that quercetin glycosides and 6-methoxy flavones have more activity than ordinary profens. The catalytic core of the enzyme is made up of three polar amino acids: serine, aspartate, and histidine.

According to scientific evidence, quercetin creates H-bonds with the polar amino acids involved in the active site of trypsin [50]. As a result, we assume that the quercetin core of the glycosides, as well as the methoxyflavones, create H-bonds, inhibiting the action of the enzyme. The high activity of quercetin glycosides is due to the fact that the quercetin core is compatible with the active center of trypsin. The following lists the anti-tryptic activities of *C. botrys* fractions in decreasing order: EtOAc > BuOH > CHCl_3_ > *n*-Hexane. This indicates that quercetin mono- and di-glycosides are more active in inhibiting trypsin, followed by quercetin triglycosides and 6-methoxy flavones.

### 2.10. Data Correlation

The data from the analysis of the fractions allow us to infer a correlation between the contents of phenols, tannins, quercetin glycosides, methoxy flavones and in vitro biological activity. Table 5 displays the information.

The oxidative stress data (HPSA and HRSA) correlate very well with the total phenolic content (−0.6916 and −0.9900) and with total tannins (−0.7183 and −0.9941) (Table 5). In general, quercetin glycosides are effective at scavenging OH radicals. Regarding nitrosative stress, the results show dependence in opposition to oxidative stress. This suggests that NOSA is affected by the structure of the flavonoid, as well as the substituents. In this particular situation, the research demonstrates that 6-methoxy flavones have a high affinity for quenching NO radicals (r = −0.8716).

The results of the study demonstrate that there is a strong relationship between in vitro biological activity, *TPC*, *TTS*, and the concentration of quercetin glycosides. They bind to the polar groups of the allosteric centers of albumin and the active center of trypsin because they are more polar than 6-methoxy flavones. The structure of biologically active components influences in vitro biological activity. There was only a modest relationship between 6-methoxy flavones and *IAD* and *ATA*. However, this aspect should not be overlooked, because there is evidence that methoxy flavones have anticancer and other properties. The in vitro techniques *IAD* and *ATA* showed a moderate correlation dependency (r = 0.6362). This can be explained by the fact that the two approaches serve the same purpose, which is to keep the albumin molecule intact. Albumin is protected against denaturation (*IAD*) in the first example. Polyphenols bind to albumin allosterically in this case. In the second situation, it is shielded from the action of the enzyme trypsin (ATA), since polyphenols inhibit the enzyme. These two mechanisms prevent plasma viscosity from changing during inflammation and the progression of arthritic disorders.

## 3. Materials and Methods

### 3.1. Chemicals and Reagents

For HPLC analysis, chromatographic-grade methanol was used (VWR, Vienna, Austria). A Millipore purifier (Millipore, Burlington, MA, USA) was used to obtain water for HPLC. Potassium dihydrogen phosphate, dipotassium hydrogen phosphate, phosphoric acid, potassium chloride, quercetin, hispiduline, ascorbic acid, ibuprofen, ketoprofen, sodium chloride, hydrogen peroxide, trypsin, Tris-HCl buffer, sodium nitroprusside, sodium salicylate, ferrous sulfate, sulfanilamide, naphthylethylenediamine dihydrochloride and perchloric acid were purchased from Sigma-Aldrich, Taufkirchen, Germany. Human albumin 20%—BB, 200 g/L was ordered from BB-NCIPD Ltd., Sofia, Bulgaria.

### 3.2. Plant Material

The specimens of *C. botrys* were collected from regions of Plovdiv, village Rogosh. The plants were gathered in August consecutively, at the full flowering stage. The plant material was identified by means of voucher specimens (05269 and 05271), which were deposited in the herbarium of Agricultural University—Plovdiv by Assoc. Prof. Koicho Koev, Ph.D. (Faculty of Biology, University of Plovdiv, Bulgaria). The aerial parts of the plants were dried at room temperature in the absence of direct sunlight. The dried material was further ground to powder, packed into multilayer paper bags, and stored in a dark room at ambient temperature.

### 3.3. Fractionation of Polyphenols with Solvents of Different Polarity

The fractionation of polyphenols with different in polarity solvents was described by Bojilov and co-workers [19]. Finely, ground pith plant material (10 g) was weighed, transferred to a 500 mL Erlenmeyer flask with a stopper and extracted with 500 mL methanol for 40 min in an ultrasonic bath. The extract was filtered through a sintered glass filter under vacuum and then the filtrate was evaporated to dryness. The dry residue was weighed, and then suspended in 100 mL of distilled water. The aqueous layer was fractionated by liquid–liquid extraction—sequentially with hexane (10 × 30 mL), chloroform (10 × 30 mL), ethyl acetate (10 × 30 mL) and butanol (10 × 30 mL). The resulting fractions were evaporated to dryness using a rotary vacuum evaporator and weighed. They were then dissolved in methanol, the concentration of each fraction being 10 µg/mL. The obtained fractions were tested in vitro for their biological activity and analyzed by HPLC-PDA and UHPLC-MS.

### 3.4. Determination of Total Phenolic Content (TPC)

The total phenolic content in crude extracts was determined with the colorimetric method using Folin–Ciocalteu’s reagent [51] with slight modifications [52]. A calibration curve was constructed using as the standard an ethanolic solution of gallic acid at concentrations between 50 and 1000 µg/mL. Briefly, 100 µL of extract or gallic acid standard was mixed with 2.4 mL distilled water, 500 µL of 0.2 M Folin–Ciocalteu’s reagent and 2 mL of 7.5% sodium carbonate (Na_2_CO_3_) solution. The tested samples were incubated for 2 h in dark at room temperature. The absorbance of the samples was measured at 765 nm with a spectrophotometer (Camspec M508, Leeds, UK), using a blank sample. The total phenolic content was expressed as mg gallic acid equivalent per gram extract (mg GAE/g Extr) based on the calibration curve.

### 3.5. Determination of Total Tannin Contain (TTC)

The total tannin content (*TTC*) in the different fractions was determined spectrophotometrically using the Folin–Ciocalteu method [53]. To determine the total tannin content, we used standard solutions of tannic acid with a concentration of 10 μg/mL to 1000 μg/mL. The analysis was carried out as follows: in a 10 mL volumetric flask, add 100 μL of tannic acid fraction or standard, 7.5 mL of distilled water, 500 μL of 2 M Folin-Ciocalteu reagent and 1 mL of 35% sodium carbonate solution (Na_2_CO_3_) and the flask is topped up with distilled water up to the mark. Test samples were mixed and incubated for 30 min in the dark at room temperature. The absorbance of the samples was measured at 700 nm with a spectrophotometer (Camspec M508, UK), against a blank. Total tannin content is expressed as mg tannic acid equivalent per gram of extract (mg TAE/g Extr).

### 3.6. Analysis of Polyphenols by HPLC-PDA

The analysis of polyphenols was done according to the method developed by Bojilov et al. [19]. The equipment used for HPLC analysis consisted of a quaternary mixer Smartline Manager 5000, pump Smartline 1000 and PDA 2800 detector (Knauer, Berlin, Germany). The column used was a Kromasil C18, 150 × 4.6 mm i.d., 5 mm particle size (Supelco, Bellefonte, PA, USA). The chromatographic separation was carried out using 0.1% trifluoroacetic acid solution in acetonitrile as solvent B. As solvent A we used a mixture of 90 parts water and 10 parts 0.1% TFA acid solution in acetonitrile with the following gradient elution program: 0–10 min, 100–90% A, 10–18 min, 89% A, 18–25 min, 85% A, 25–40 min, 45% A. The polyphenols were monitored at 280 nm, 320 nm, 340 nm and 352 nm. The mobile phase flow rate was set by 1.0 mL/min; the sample volume was 20 μL. Quantification of quercetin glycosides and 6-methoxy flavones was done by the external standard method using quercetin and hispidulin as standard substances. The correlation coefficients for quercetin (Y = 67.7x + 16) and hispidulin (Y = 105.8x + 27) were 0.9994 and 0.9996, respectively.

### 3.7. Identification of Flavonoids by Orbitrap UHPLC-MS/MS

The analysis of polyphenols with UHPLC-MS/MS was accomplished according to the method developed by Bojilov et al. [19]. The UHPLC analysis was performed on a Thermo UHPLC (Q-Exactive, Thermo Fisher Scientific, Waltham, MA, USA) system piloted by Xcalibur software. The chromatographic separation was achieved on a column SunShel C18, 150 × 2.1 mm i.d., 2.6 µm particle size (ChromaNik Technologies Inc., Osaka City, Japan). As a mobile phase for solvent A we used a mixture of 90 parts 2% formic acid in water and 10 parts solvent B 2% formic acid in acetonitrile. The gradient elution was set as follows: 0–1 min, 90% A; 1–15 min, 70% A, 15–22 min 50% A, 22–28 min 0% A, maintained up to 34 min by 0% A. The flow rate was 200 µL/min and the injection volume was 2 µL. The system was equipped with an ESI source that was operated in negative mode under the following conditions: sheath gas flow rate 30 L/min, aux gas flow rate 8 L/min, sweep gas flow rate 4 L/min, spray voltage 3.5 kV, capillary temp. 350 °C, S-lens RF level 50, 250 °C heater temperature. Nitrogen was used as the collision gas. Optimization of the normalized collision energy (NCE) was performed in the range of 20 eV to 55 eV for negative mode. The collision-induced dissociation (CID) was 10 eV. The spectra were scanned in the range of *m*/*z* 100–1000 with a resolution of 35,000. The identification of flavonoids was made on the basis of two scan events: full scan MS mode and MS/MS experiment, and by comparing the chromatographic and the mass spectral data of the investigated compounds with those of rutin, quercetin 3-*β*-D-glucoside, quercetin 3-*β*-D-galactoside, quercetin, and hispidulin.

### 3.8. Methods for Investigation of Biological Activity

#### 3.8.1. Hydrogen Peroxide Scavenging Activity (HPSA)

The Manolov et al. approach was used to evaluate the capacity to scavenge hydrogen peroxide [54]. A 43 mM solution of H_2_O_2_ was prepared in potassium phosphate buffer solution (0.2 M, pH 7.4). The analysis of the samples was carried out as follows: in test tubes, 0.6 mL H_2_O_2_ (43 mM), 1 mL sample/standard with different concentrations (20–1000 µg/mL), and 2.4 mL potassium phosphate buffer solution were mixed. The mixture was stirred and incubated in the dark for 10 min at 37 °C. Absorbance was measured at 230 nm with a spectrophotometer (Camspec M508, Leeds, UK) against a blank solution containing phosphate buffer and H_2_O_2_ without the sample. Ascorbic acid and quercetin were used as standards. The percentage HPSA of the samples was evaluated by comparing with a blank sample and calculated using the following formula:

(1) $$I,\%(HPSA)=\left[\frac{A_{blank}-(A_{TS}-A_{CS})}{A_{blank}}\right]\times 100$$

where *A_blank_* is the absorbance of the blank sample, *A_CS_* is the absorbance of the control sample, and *A_TS_* is the absorbance of the test sample.

#### 3.8.2. Hydroxyl Radical Scavenging Activity (HRSA)

Hydroxyl radical scavenging activities of different fractions of *C. botrys* were determined according to the method described by Guo [55]. The scavenging of hydroxyl radicals was done as follows: 0.3 mL sodium salicylate (20 mM), 1 mL FeSO_4_ (1.5 mM), 1 mL sample/standard with different concentrations (20–1000 µg/mL), and 0.7 mL H_2_O_2_ (6 mM). They were mixed immediately, and then the reaction tubes were put in a 37 °C water bath for 1 h, and the absorbance of the mixture was recorded at 510 nm against a blank. We used standards with proven high antioxidant activity, such as ascorbic acid and quercetin. The hydroxyl radical scavenging ability was calculated as follows:

(2) $$I,\%(HRSA)=\left[\frac{A_{blank}-A_{sample}}{A_{blank}}\right]\times 100$$

where *A_blank_* is the absorbance without samples and *A_sample_* is the absorbance in the presence of the samples of different fractions from *C. botrys*.

#### 3.8.3. Nitric Oxide Scavenging Activity (NOSA)

NO inhibition was measured according to the method of Marcocci [56]. Sodium nitroprusside (SNP) was used as the source for generating NO. The SNP solution (5 mM) was prepared in phosphate-buffered saline (PBS, 0.2 M, pH 7.4). Briefly, the reaction mixture containing 0.5 mL SNP (5 mM) and 1 mL sample/standard with different concentration (15–1000 μg/mL) was incubated at 25 °C for 180 min. At the end of the reaction time, an equal amount of Griess reagent (1% sulfanilamide in 2% phosphoric acid and 0.1% naphthylethylenediamine dihydrochloride) was added. The absorbance of the chromophore (purple azo dye) formed during the diazotization of nitrite ions with sulfanilamide and subsequent coupling with naphthylethylenediamine dihydrochloride was measured at 546 nm. Quercetin and ascorbic acid were used as standards. The NO uptake capacity was calculated as follows:

(3) $$I,\%(NOSA)=\left[\frac{A_{blank}-A_{sample}}{A_{blank}}\right]\times 100$$

where *A_blank_* is the absorbance of a blank and *A_sample_* is the absorbance of the test sample.

#### 3.8.4. Inhibition of Albumin Denaturation (IAD)

The in vitro anti-inflammatory activity was assessed via the inhibition of albumin denaturation (*IAD*). The analysis was performed according to the Manolov method [57] with minor modification. The experiment was performed with human albumin. The solution of albumin (1%) was prepared in distilled water (pH 7.4). The tested compounds and standard were dissolved first in PBS so the final concentration of the stock solution was 1000 μg/mL. Then, a series of working solutions with different concentrations (20–500 μg/mL) in PBS were prepared. The reaction mixture contained 2 mL of test sample/standard of different concentrations and 1 mL albumin (1%). The mixture was incubated at 37 °C for 15 min and then heated at 70 °C for 15 min in the water bath. After cooling, the turbidity was measured at 660 nm with a spectrophotometer (Camspec M508, Leeds, UK). Ibuprofen and ketoprofen were used as standards. The experiment was performed three times. The percentage inhibition of albumin denaturation (*IAD*) was calculated against control. The control sample was albumin with the same concentration dissolved in distilled water.

(4) $$\%IAD=\left[\frac{A_{blank}-A_{sample}}{A_{blank}}\right]\times 100$$

#### 3.8.5. Antitryptic Activity (ATA)

This method is known also as in vitro anti-arthritic activity. The analysis was performed according to the method of Oyedapo and Femurewa [49] with minor modification as described by Manolov et al. [57]. The reaction mixture contained 2 mL 0.06 mg/mL trypsin, 1 mL Tris-HCl buffer (20 mM, pH 7.4) and 1 mL test sample/standard (in methanol) of different concentrations (20–1000 μg/mL). The mixture was incubated at 37 °C for 5 min. Then 1 mL of human albumin (4% *v*/*v*) was added. The mixture was incubated for an additional 20 min. To the mixture, 2 mL of 70% perchloric acid was added for termination of the reaction. The cloudy suspension was cooled and centrifuged at 5000 rpm for 20 min. The absorbance of the supernatant was measured at 280 nm with a spectrophotometer (Camspec M508, Leeds, UK) against a control solution. The control solution was the sample/standard in methanol with different concentrations. Ibuprofen and ketoprofen were used as standards. The analysis was performed three times. The percentage of antitryptic activity (ATA) of the samples was evaluated by comparing with a blank sample. The blank sample was prepared as the test sample but with a small exception—perchloric acid was added before albumin.

(5) $$\%ATA=\left[\frac{A_{blank}-\left(A_{TS}-A_{CS}\right)}{A_{blank}}\right]\times 100$$

where *A_blank_* is the absorbance of the blank sample, *A_CS_* is the absorbance of the control solution (test sample in different concentrations) and *A_TS_* is the absorbance of the test samples.

### 3.9. Statistical Analysis

All the analyses were made in triplicates. Data were expressed as mean ± SD. A statistical SPSS 19.0 software package (SPSS Inc., Chicago, IL, USA) was used. The level of significance was set at *p* < 0.05. The mean IC_50_ value was estimated based on three replicates by interpolating the graphical dependence of activity on concentration.

## 4. Conclusions

The aim of this study was to investigate the polyphenolic complex of *C. botrys*, which was fractionated with differences in polarity solvents and analyzed by HPLC-PDA and UHPLC-MS in negative mode. The flavonoids pectolinarigenin, demethylnobiletin and isosinensetin, and glycosides of quercetin (triglycosides, acylglycosides), kaempferol, isorhamnetin, hispidulin, and jaceosidine, were identified and reported in *Chenopodium botrys* for the first time. By using HPLC analysis, we have discovered that the primary components of the glycoside group are quercetin glycosides in a small amount, while the content of 6-methoxy flavones is the highest. The biological activity of the individual fractions was investigated. The applied approach allows us to distinguish the polyphenolic components via their biological activity.

In general, glycosides and 6-methoxy flavones have high biological activity (*HPSA*, *HRSA*, *NOSA*, *IAD*, and *ATA*). We may derive valuable knowledge on the characteristics of the physiologically active components present in *C. botrys* from the experimental results. Aerobic organisms can be shielded from the negative effects of ROS by quercetin mono- and di-glycosides, which are potent oxidants. 6-Methoxy flavones, on the other hand, are more effective in scavenging NO radicals.

Quercetin glycosides and 6-methoxy flavones are characterized by high in vitro anti-arthritic activity assessed by ATA, but only mono- and di-glycosides demonstrate high in vitro anti-inflammatory activity. As a result, fractionation is an important stage of the analysis, in which the components are separated based on polarity, and each class of molecules may more accurately reveal its medicinal and pharmacological purpose. The HPSA, HRSA, and NOSA data give more than just information on the antioxidant capacity of the fractions. Polyphenols can also be employed as anti-inflammatory drugs, since it has been shown that the creation of ROS is enhanced during an inflammatory process, while 6-MFs with high NOSA may be useful in reducing the harm done by ischemia–reperfusion to inducible NOS (iNO synthase) function [58,59].

## Figures and Tables

**Table 1:** Yield, total phenolic content (TPC), total tannin content (TTC), amount of quercetin-glycosides (Qrc-Glc) determined by HPLC-DAD, and 6-methoxy flavones (6-MFs) determined by HPLC-DAD.

Fractions	Yield	TPC	TTC	Qrc-Glc	6-MFs
	%	mgGAE/g Extr	mgTAE/g Extr	mg/g Extr	mg/g Extr
***n*** **-H**	33.02 ± 0.71	18.41 ± 1.76	10.40 ± 0.79	-	-
**CHCl_3_**	12.30 ± 0.71	48.92 ± 2.26	36.97 ± 3.05	-	355.47 ± 7.14
**EtOAc**	10.74 ± 0.94	159.84 ± 2.59	133.08 ± 14.56	168.82 ± 3.73	-
**BuOH**	24.93 ± 1.17	50.05 ± 2.06	33.85 ± 1.41	67.21 ± 1.56	-

**Table 2:** ESI-MS/MS product ions obtained from the [M−H]^−^ ions of 6-methoxyflavones in *C. botrys*. ^a^ error; ^b^ *m*/*z* (relative abundance).

	Nepetin	Hispidulin	Jaceosidin	Eupatilin	Pectolinarigenin
[M–H]^−^	315.0515 [−3.17] ^a^	299.0573 [−5.68]	329.0671 [−3.04]	343.0831 [−3.79]	313.0732 [4.47]
[M–H–CH_3_]^−^	300.0275 (22.4) ^b^	284.0331 (20.4)	314.0437 (2.6)	328.0598 (1.2)	-
[M–H–CH_4_]^−^	-	-	313.0363 (3.1)	-	-
[M–H–2CH_3_]^−^	-	-	299.0207 (16.4)	313.0364 (37.6)	283.0254 (80.24)
[M–H–CH_3_–HCO]^−^/[M–2H–CH_3_–CO]^−^	271.0243 (2.8)	255.0301 (4)	285.0412 (2)	-	-
[M–H–3CH_3_]^−^	-	-	-	298.0127 (100)	-
[M–H–2CH_3_–CO]^−^	-	-	271.0251 (100)	285.0413 (14.3)	255.0301 (77.31)
[M–H–3CH_3_–CO]^−^	-	-	-	270.0174 (65.7)	-
[M–2H–CH_3_–CO_2_]^−^	255.0299 (2.8)	-	-	-	-
[M–H–3CH_3_–CO_2_]^−^	-	-	-	254.0223 (3.6)	-
[M–H–2CH_3_–2CO]^−^	-	-	243.0299 (6.8)	-	227.0347 (17.32)
[M–H–3CH_3_–2CO]^−^	-		-	242.0221 (27.7)	-
[M–2H–CH_3_–2CO]^−^	243.0301 (5.3)	227.0347 (14.6)	-	-	-
[M–H–2CH_3_–CO–CO_2_]^−^	-	-	227.0347 (5.7)	-	211.0401 (12)
[M–H–3CH_3_–CO–CO_2_]^−^	-		-	226.0269 (8.9)	-
[M–H–CH_3_–C_2_H_2_O–CO]^−^	-	214.0267 (1)	-	-	-
[M–2H–CH_3_–CO–CO_2_]^−^	227.0348 (9.7)	211.0391 (14.3)	-	-	-
[M–H–2CH_3_–3CO]^−^	–	-	215.0345 (4.6)	-	199.0392 (3.5)
[M–H–3CH_3_–3CO]^−^	-		-	214.0267 (11.8)	-
[M–2H–CH_3_–3CO]^−^	215.0343 (4.6)	199.0394 (7.7)	-	-	-
[M–H–2CH_3_–2CO–CO_2_]^−^	-	-	199.0395 (16.7)	-	183.0442 (33)
[M–H–3CH_3_–2CO–CO_2_]^−^	-		-	198.0315 (13.4)	-
[M–2H–CH_3_–2CO–CO_2_]^−^	199.0392 (15.7)	183.0439 (11)	-	-	-
[M–H–2CH_3_–4CO]^−^	-	-	187.0391 (2.3)	-	-
[M–H–3CH_3_–4CO]^−^	-		-	186.0317 (2.5)	-
[M–H–2CH_3_–3CO–CO_2_]^−^	-	-	171.0441 (5.4)	-	-
[M–H–3CH_3_–3CO–CO_2_]^−^	-		-	170.0363 (5.1)	-
CH_3_^1.3^A^−^	165.9896 (3.5)	165.9894 (2.7)	165.9899 (1.7)	165.9900 (3.7)	165.9894 (4)
[CH_3_^1.3^A–H]^−^	164.9818 (2.2)	-	164.9818 (2.2)	164.9818 (11.2)	164.9817 (12.53)
[CH_3_^0.2^A^−^]/[M–H–CH_3_–HCO–ring B]^−^	163.0026 (5)	-	163.0026 (2.6)	163.0025 (34)	163.0025 (72)
[CH_3_^1.3^A–CO]^−^	136.9866 (63)	136.9866 (84)	136.9866 (12)	136.9866 (16.8)	136.9866 (20.02)
[CH_3_^0.2^A^−^–CO]/[M–H–CH_3_–HCO–ring B–CO]^−^	-	-	135.0037 (2)	135.0037 (28)	135.0037 (40)
^1.3^B^−^	133.0290 (17.8)	117.0330 (8.2)	147.0439 (3.2)	-	-
CH_3_^1.3^B^−^ + H	-	-	133.0281 (9.8)	-	-
CH_3_^1.3^B^−^	-	-	132.0202 (1)	147.0438 (30.5)	117.0331 (67.66)
2CH_3_^1.3^B^−^	-	-	-	132.0202 (46)	-
[CH_3_^1.3^A–2CO–CO_2_]^−^	65.0016 (100)	65.0016 (100)	65.0016 (25)	65.0015 (16)	65.0016 (28.18)

**Table 3:** Retention time, molecular ion and fragment ions (UHPLC-MS/MS) of glycosides identified in the ethyl acetate and butanol fractions of *C. botrys*.

Fraction	Peak №	Name	UHPLC-MS	[M–H]^−^	MS/MS
			t_R_	(Δm, ppm)	
BuOH fraction	1	Quercetine 3-*O*-(2″-*O*-apiofuranosyl-6″-*O*-glucopyranosyl)- β-D-glucopyranoside	12.11	757.1839 (0.79)	625, 463, 300, 271, 255, 243, 179, 151, 112, 121, 107
	2	Quercetine 3-*O*-(2″,6″-di-*O*-α-L-rhamnopyranosyl)-β-D- galactopyranoside (acyl: malonyl)	12.36	841.2052 (0.95)	463, 300, 271, 255, 243, 179, 151, 121, 112, 107
	3	Quercetine 3-*O*-(2″,6″-di-*O*-α-L-rhamnopyranosyl)- β-D-glucopyranoside (acyl: malonyl)	12.70	841.2052 (0.95)	463, 300, 271, 255, 243, 179, 151, 121, 112, 107
	4	Quercetine 3-*O*-α-L-rhamnopyranosyl-α-L-rhamnopyranosyl- β-D-malonylglucopyranoside	13.69	841.2052 (0.95)	463, 300, 271, 255, 243, 179, 151, 121, 112, 107
	5	Quercetine 3-*O*-α-L-rhamnopyranosyl-α-L-rhamnopyranosyl- β-D-malonylglucopyranoside (acyl)	13.77	883.2162 (1.02)	841, 737, 463, 300, 271, 255, 243, 179, 151, 121, 107
EtOAc fraction	6	Rutin	12.14	609.1467 (0.98)	300, 271, 255, 243, 179, 151, 107
	7	Quercetin-*O*-pentoside-hexoside	12.22	595.1310 (0.84)	300, 271, 255, 243, 179, 151, 107
	8	Quercetin-*O*-(acyl)-hexoside-hexoside	12.31	667.1524 (1.20)	625, 463, 300, 271, 255, 243, 179, 151, 107
	9	Quercetin-*O*-(acyl)-hexoside-(acyl)-hexoside	12.48	709.1622 (0.00)	667, 639, 505, 463, 300, 271, 255, 243, 179, 151, 107
	10	Isorhamnetin 3-*O*-glucopyranoside-7-*O*-rhamnopyranoside	12.56	623.1628 (−1.60)	461, 314, 300, 285, 271, 255, 243, 227, 165, 151, 137, 125, 107
	11	Quercetin 3-*O*-galactopyranoside	12.81	463.0881 (−0.22)	301, 300, 271, 255, 243, 179, 151, 107
	12	Quercetin 3-*O*-glucopyranoside	12.90	463.0881 (−0.22)	301, 300, 271, 255, 243, 179, 151, 107
	13	Isorhamnetin 3-*O*-glucopyranoside-rhamnopyranoside	13.92	623.1628 (−1.60)	477, 315, 300, 285, 271, 255, 243, 227, 165, 151, 137, 125, 107
	14	Jaceosidin 7-*O*-glucopyranosid	13.94	491.1197 (0.41)	328, 313, 300, 285, 269, 241, 213, 164, 147, 133
	15	Quercetin-*O*-(acyl)-hexoside-(acyl)-hexoside	14.01	709.1613 (−1.27)	667, 463, 300, 271, 255, 243, 179, 151, 107
	16	Isorhamnetin 3-*O*-glucopyranoside	14.26	477.1035 (−0.63)	315, 314, 299, 285, 271, 257, 243, 227, 215, 199, 151, 125, 107
	17	Hispidulin 7-*O*-glucopyranoside	14.6	461.1084 (−1.08)	297, 283, 269, 255, 227, 183, 164, 163, 136, 135, 117
	18	Kaempferol-*O*-(acyl)-hexoside-(acyl)-hexoside	14.85	693.1680 (1.15)	489, 447, 285, 255, 227, 213, 151, 125, 107
	19	Jaceosidin 4′-*O*-glucopyranosid	14.94	491.1191 (−0.81)	328, 313, 298, 285, 270, 161, 164, 163, 136, 135, 132
	20	Quercetin-glycoside—unidentified	15.03	883.2127	300, 271, 255, 243, 179, 151, 107
	21	Quercetin-glycoside—unidentified	15.71	853.2035	300, 271, 255, 243, 227, 151, 107
	22	Kaempferol-glycoside—unidentified	15.88	735.1791	443, 285, 284, 255, 227, 213, 151, 121, 125, 107
	23	Quercetin-glycoside—unidentified	16.22	751.1723	300, 271, 255, 243, 227, 179, 151, 121, 107
	24	Quercetin-glycoside—unidentified	16.40	751.1740	300, 271, 255, 243, 227, 179, 151, 121, 107
	25	Quercetin-glycoside—unidentified	16.82	925.2233	300, 271, 255, 243, 179, 151, 107
	26	Quercetin-glycoside—unidentified	17.67	793.1818	300, 271, 255, 243, 179, 151, 121, 107

**Table 4:** Hydrogen peroxide scavenging activity (HPSA), hydroxyl radical scavenging activity (HRSA), nitric oxide scavenging activity (NOSA), inhibition of albumin denaturation (IAD) and anti-tryptic activity (ATA). Ascorbic acid (AA), quercetin (Qrc), ibuprofen (Ibu) and ketoprofen (Ket) were used as standards.

Fractions	HPSA	HRSA	NOSA	IAD	ATA
	IC_50_ ± SD, μg/mL				
***n*** **-H**	511.49 ± 33.33	149.57 ± 2.02	212.45 ± 21.76	945.06 ± 86.88	560.99 ± 15.24
**CHCl_3_**	417.03 ± 34.07	139.32 ± 8.85	146.59 ± 14.94	840.38 ± 60.03	202.44 ± 23.36
**EtOAc**	39.18 ± 3.26	105.03 ± 3.52	185.25 ± 7.63	461.41 ± 43.84	116.23 ± 11.59
**BuOH**	978.07 ± 136.03	143.76 ± 3.46	227.12 ± 26.61	848.38 ± 68.93	145.56 ± 4.73
**Standard**					
**AA**	24.84 ± 0.35	129.84 ± 4.52	184.11 ± 10.78	-	-
**Qrc**	69.25 ± 1.82	149.18 ± 9.27	64.82 ± 0.74	-	-
**Ibu**	-	-	-	81.50 ± 4.95	259.82 ± 9.14
**Ket**	-	-	-	126.58 ± 5.00	720.57 ± 19.78

**Table 5:** Correlation between total phenolic content (TPC), total tannins (TTS), content of quercetin glycosides (Qrc-Glc), methoxy flavones (6-MFs), antioxidant activity (HPSA), hydroxyl radical scavenging activity (HRSA), nitric oxide scavenging activity (NOSA), inhibition of albumin denaturation (IAD), and anti-tryptic activity (ATA) expressed as r.

	*TTC*	*Qrc-Glc*	*6-MFs*	*HPSA*	*HRSA*	*NOSA*	*IAD*	*ATA*
***TPC***	0.9993	0.9412	−0.2177	−0.6916	−0.9900	−0.2133	−0.9996	−0.6511
***TTC***	1	0.9306	−0.2038	−0.7183	−0.9941	−0.2356	−1.0000	−0.6312
***Qrc-Glc***		1	−0.4931	−0.4596	−0.8853	0.1204	−0.9336	−0.6299
***6-MFs***			1	−0.1199	0.1139	−0.8716	0.2078	−0.1741
***HPSA***				1	0.7773	0.5843	0.7115	0.0819
***HRSA***					1	0.3343	0.9931	0.6126
***NOSA***						1	0.2296	0.2618
***IAD***							1	0.6362
***ATA***								1

## Data Availability

The data presented in this study are available in this article and in the supporting Supplementary Material.

## Supplementary Materials

The following supporting information can be downloaded at: https://www.mdpi.com/article/10.3390/molecules28124816/s1. Figure S1: Mass spectrum of hispidulin obtained by negative ion ESI-MS/MS; Figure S2: Proposed fragmentation of deprotonated hispidulin [M–H]^−^ at CE 55 eV; Figure S3: Mass spectrum of jaceosidin obtained by negative ion ESI-MS/MS; Figure S4: Proposed fragmentation of deprotonated jaceosidin [M–H]^−^ at CE 55 eV; Figure S5: Mass spectrum of eupatilin obtained by negative ion ESI-MS/MS; Figure S6: Proposed fragmentation of deprotonated eupatilin [M–H]^−^ at CE 55 eV; Figure S7: Mass spectrum of pectolinarigenin obtained by negative ion ESI-MS/MS; Figure S8: Proposed fragmentation of deprotonated pectolinarigenin [M–H]^−^ at CE 55 eV; Figure S19: Mass spectrum of sinensetin and sinensetin isomer obtained by negative ion ESI-MS/MS; Figure S10: Proposed fragmentation of deprotonated sinensetin [M–H]^−^ at CE 55 eV; Figure S11: Mass spectrum of sinensetin and sinensetin isomer obtained by positive ion ESI-MS/MS; Figure S12: Proposed fragmentation of protonated sinensetin [M + H]+ at CE 60 eV; Figure S13: Mass spectrum of nobiletin obtained by negative ion ESI-MS/MS; Figure S14: Proposed fragmentation of deprotonated nobiletin [M–H]^−^ at CE 55 eV; Figure S15: Mass spectrum of demethylnobiletin obtained by negative ion ESI-MS/MS; Figure S16: Mass spectrum of rutin obtained by negative ion ESI-MS/MS; Figure S17: Total ion current (TIC) of flavonoids from *C. botrys* in EtOAc fraction (A); Profile of selected isorhamnetin glycosides (B); Profile of selected jaceosidin glycosides (C); Profile of selected hispidulin glycoside (D).
