# Supplementary material for: Characterization of Polyphenols from Chenopodium botrys after Fractionation with Different Solvents and Study of Their In Vitro Biological Activity
Source: Molecules. 2023 Jun 16;28(12):4816. doi: 10.3390/molecules28124816 (PMC10300873; doi:10.3390/molecules28124816)
Supplement: Supplementary file 1 [file molecules-28-04816-s001.zip › molecules-2433854 - Supplimentary-material.pdf]

Supplementary Materials:

Characterization of polyphenols from *Chenopodium botrys* after fractionation with different solvents and study of their in vitro biological activity

Dimitar Bojilov *, Stanimir Manolov, Angelika Nacheva, Soleya Dagnon and Iliyan Ivanov

Contents

Figure S1. Mass spectrum of hispidulin obtained by negative ion ESI-MS/MS 2

Figure S2. Proposed fragmentation of deprotonated hispidulin $[M-H]^-$ at CE 55 eV. 2

Figure S3. Mass spectrum of jaceosidin obtained by negative ion ESI-MS/MS 3

Figure S4. Proposed fragmentation of deprotonated jaceosidin $[M-H]^-$ at CE 55 eV. 3

Figure S5. Mass spectrum of eupatilin obtained by negative ion ESI-MS/MS 4

Figure S6. Proposed fragmentation of deprotonated eupatilin $[M-H]^-$ at CE 55 eV. 4

Figure S7. Mass spectrum of pectolinarigenin obtained by negative ion ESI-MS/MS.....5

Figure S8. Proposed fragmentation of deprotonated pectolinarigenin $[M-H]^-$ at CE 55 eV. 5

Figure S9. Mass spectrum of sinensetin and sinensetin isomer obtained by negative ion ESI-MS/MS .6

Figure S10. Proposed fragmentation of deprotonated sinensetin $[M-H]^-$ at CE 55 eV. 6

Figure S11. Mass spectrum of sinensetin and sinensetin isomer obtained by positive ion ESI-MS/MS.7

Figure S12. Proposed fragmentation of protonated sinensetin $[M+H]^+$ at CE 60 eV. 7

Figure S13. Mass spectrum of nobiletin obtained by negative ion ESI-MS/MS 8

Figure S14. Proposed fragmentation of deprotonated nobiletin $[M-H]^-$ at CE 55 eV. 8

Figure S15. Mass spectrum of demethylnobiletin obtained by negative ion ESI-MS/MS 9

Figure S16. Mass spectrum of rutin obtained by negative ion ESI-MS/MS 9

Figure S17. Total ion current (TIC) of flavonoids from *C. botrys* in EtOAc fraction (**A**); Profile of selected isorhamnetin glycosides (**B**); Profile of selected jaceosidin glycosides (**C**); Profile of selected hispidulin glycoside (**D**); 10

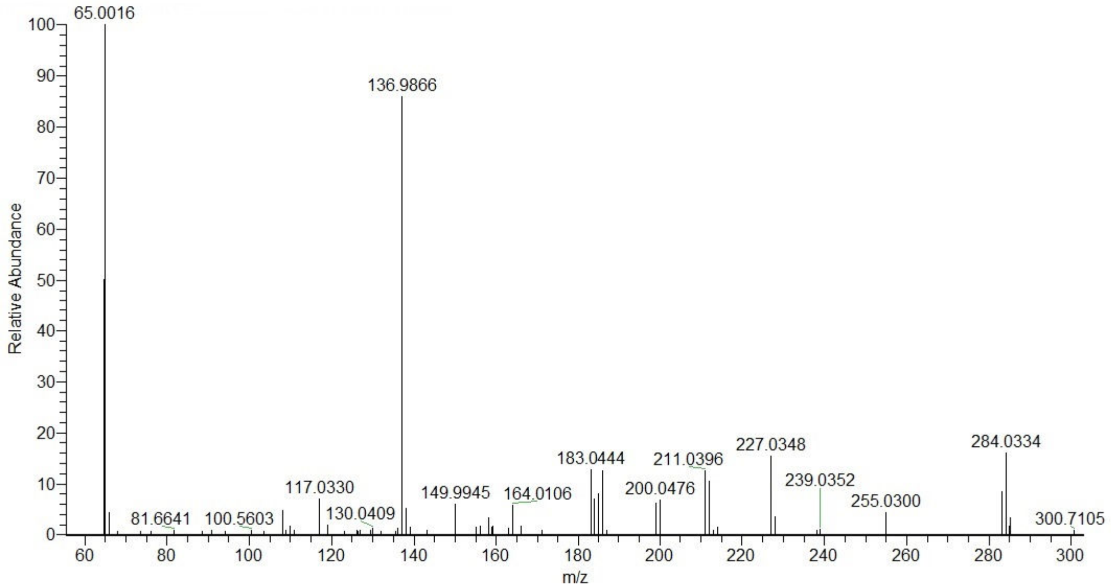

Figure S1. Mass spectrum of hispidulin obtained by negative ion ESI-MS/MS.

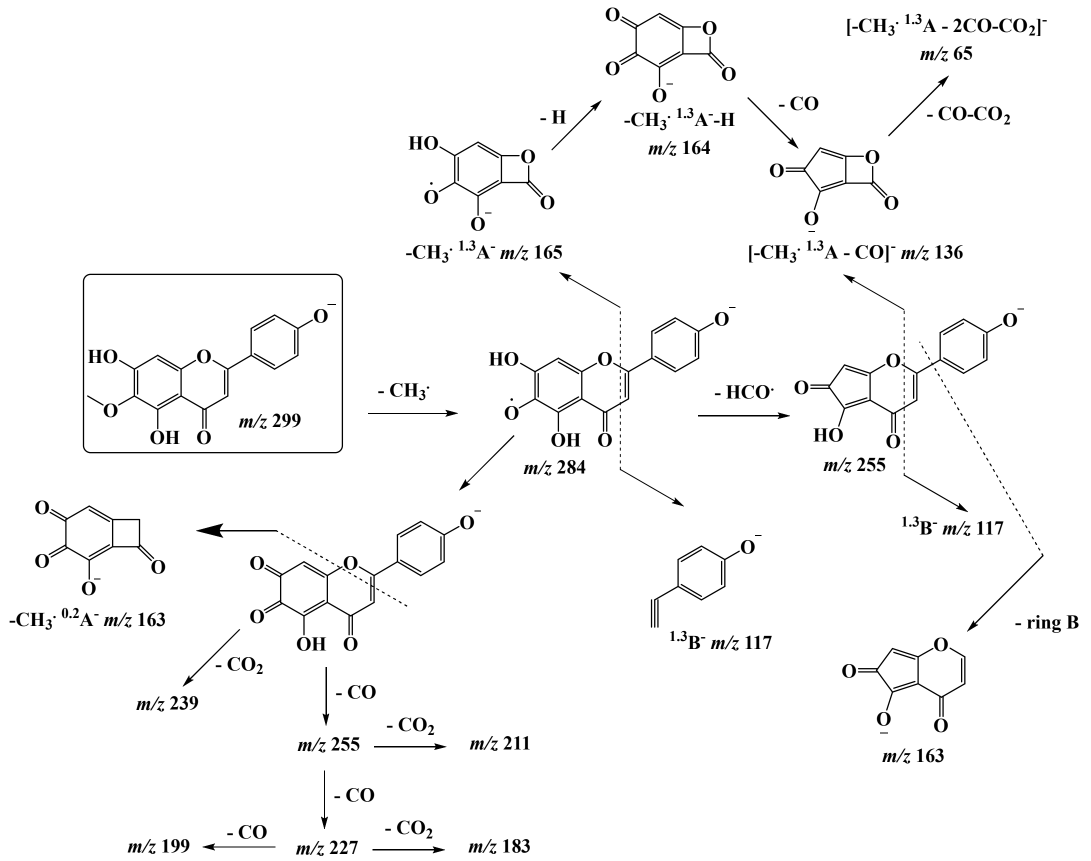

Figure S2. Proposed fragmentation of deprotonated hispidulin [M-H]⁻ at CE 55 eV.

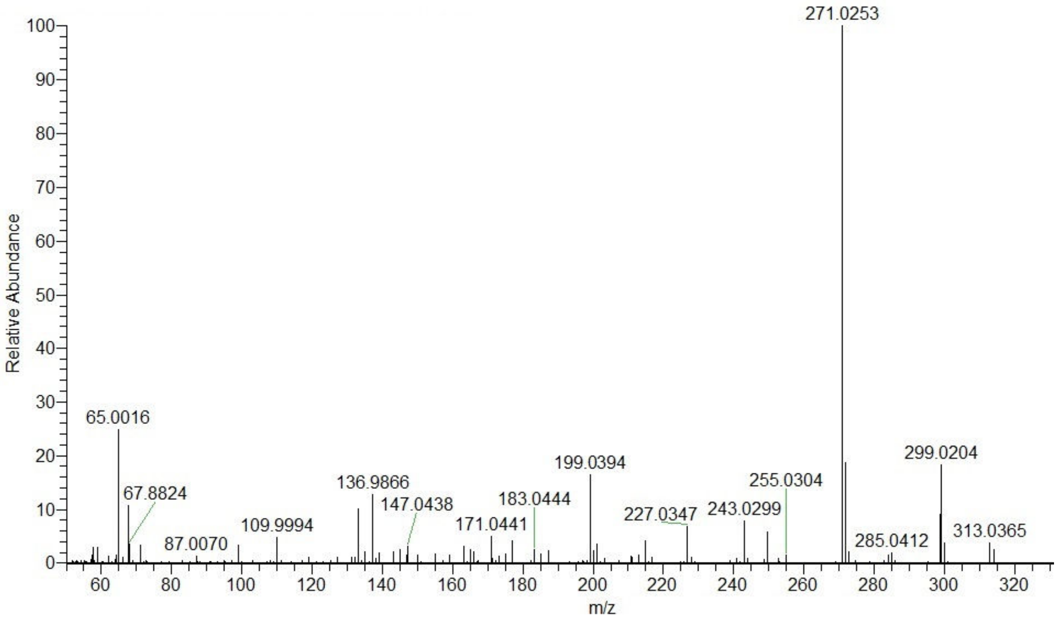

Figure S3. Mass spectrum of jaceosidin obtained by negative ion ESI-MS/MS.

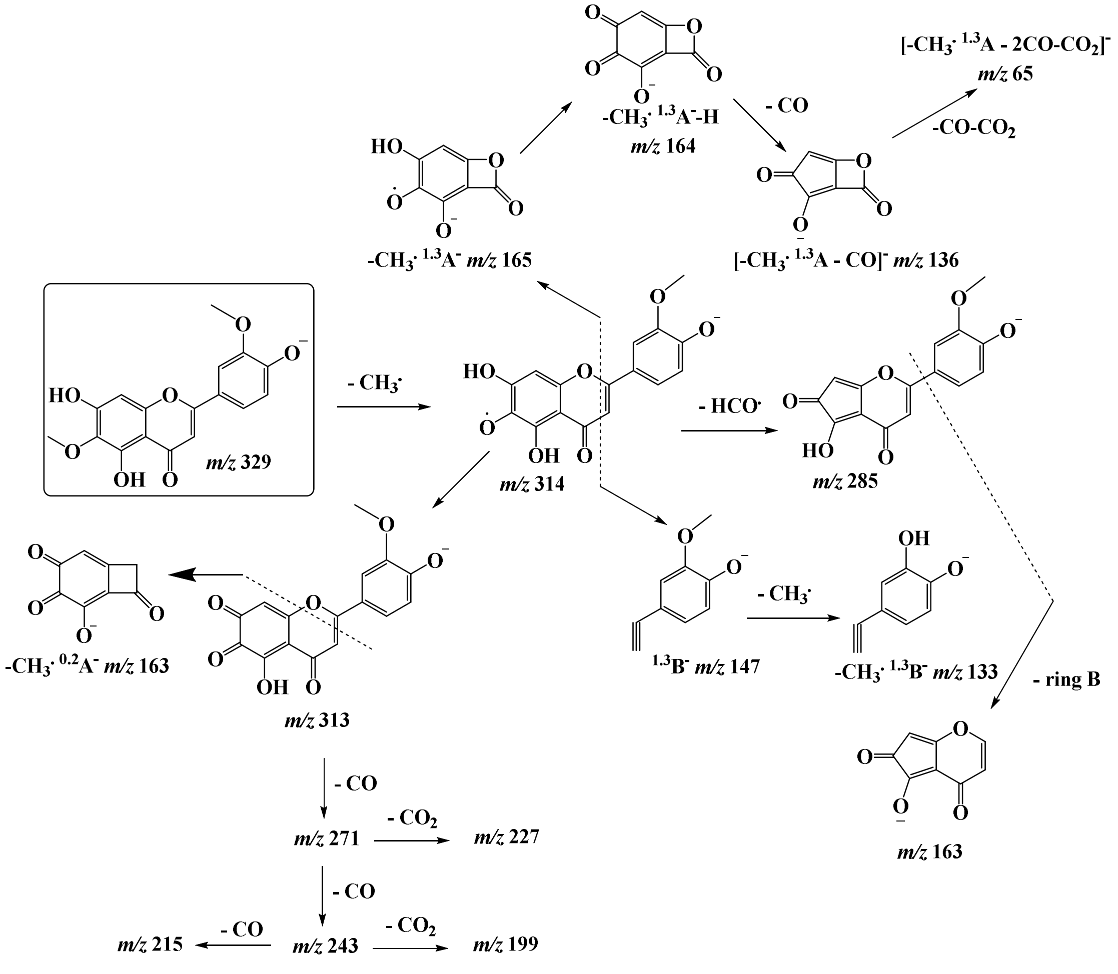

Figure S4. Proposed fragmentation of deprotonated jaceosidin [M-H]⁻ at CE 55 eV.

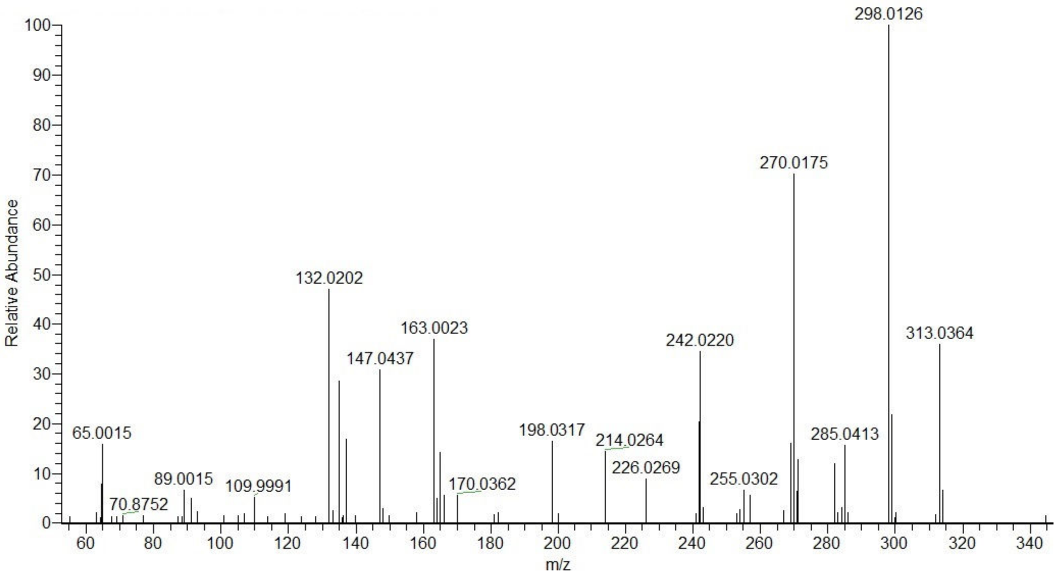

Figure S5. Mass spectrum of eupatilin obtained by negative ion ESI-MS/MS.

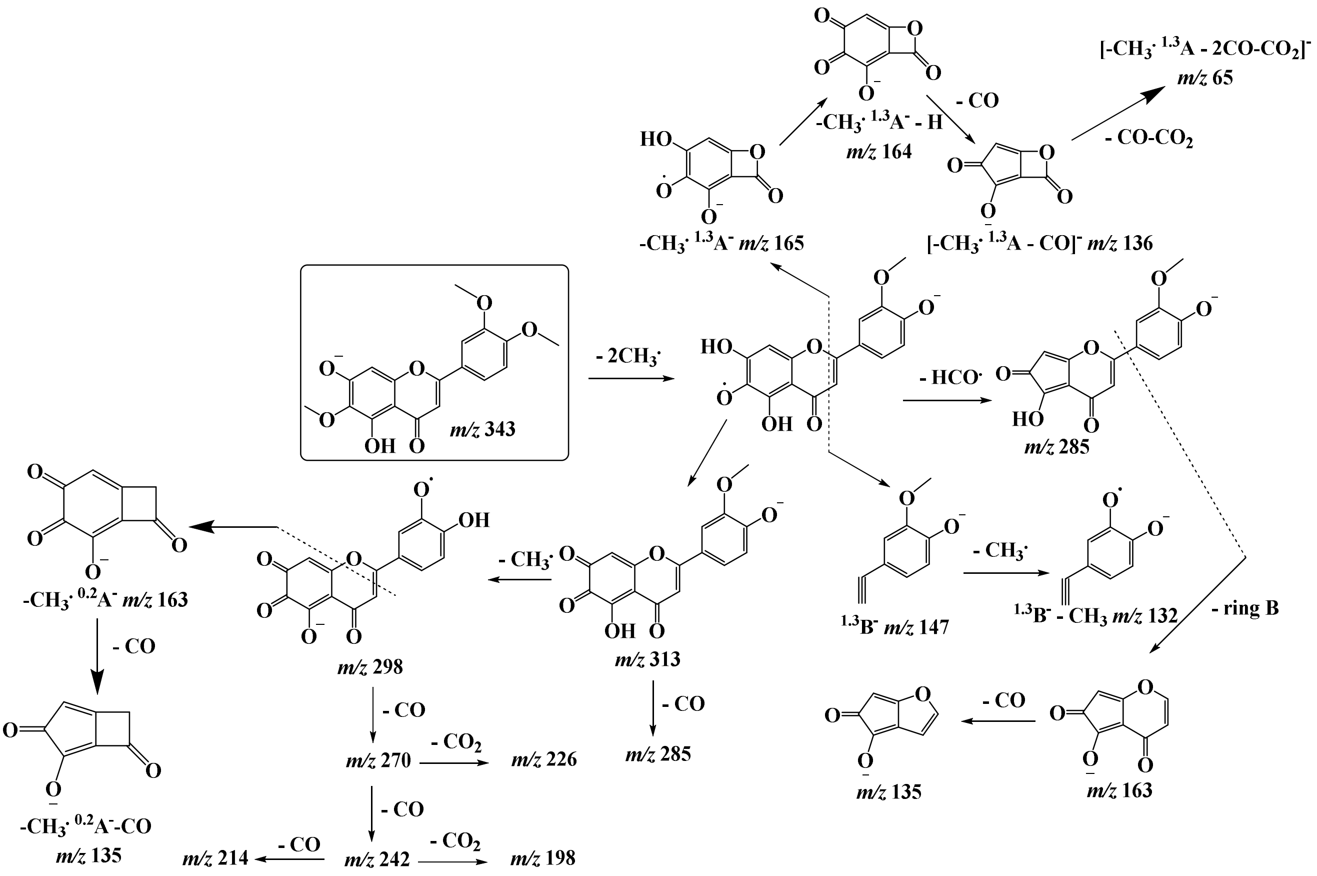

Figure S6. Proposed fragmentation of deprotonated eupatilin [M-H]⁻ at CE 55 eV.

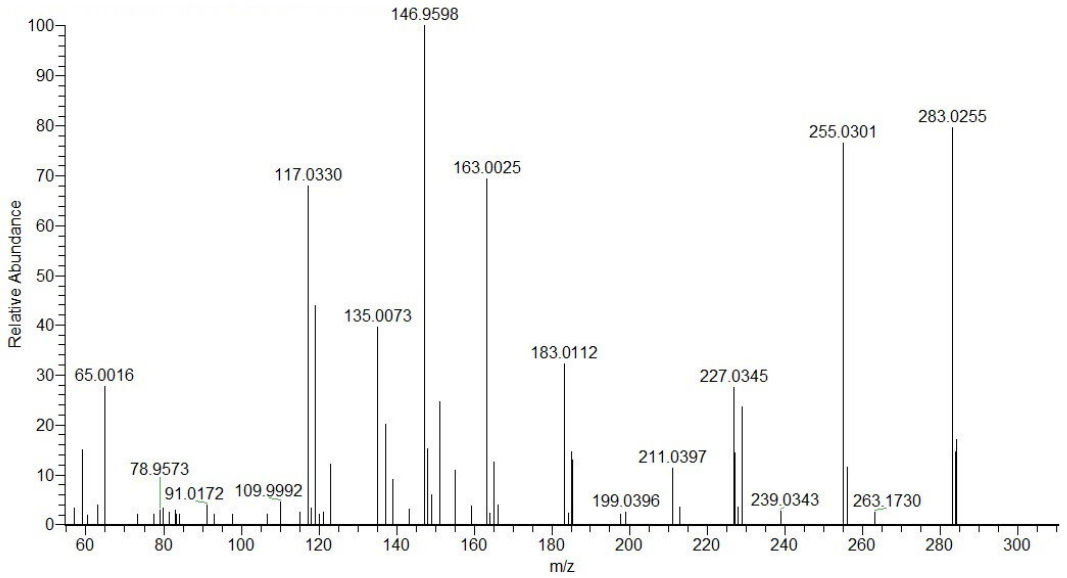

Figure S7. Mass spectrum of pectolinarigenin obtained by negative ion ESI-MS/MS.

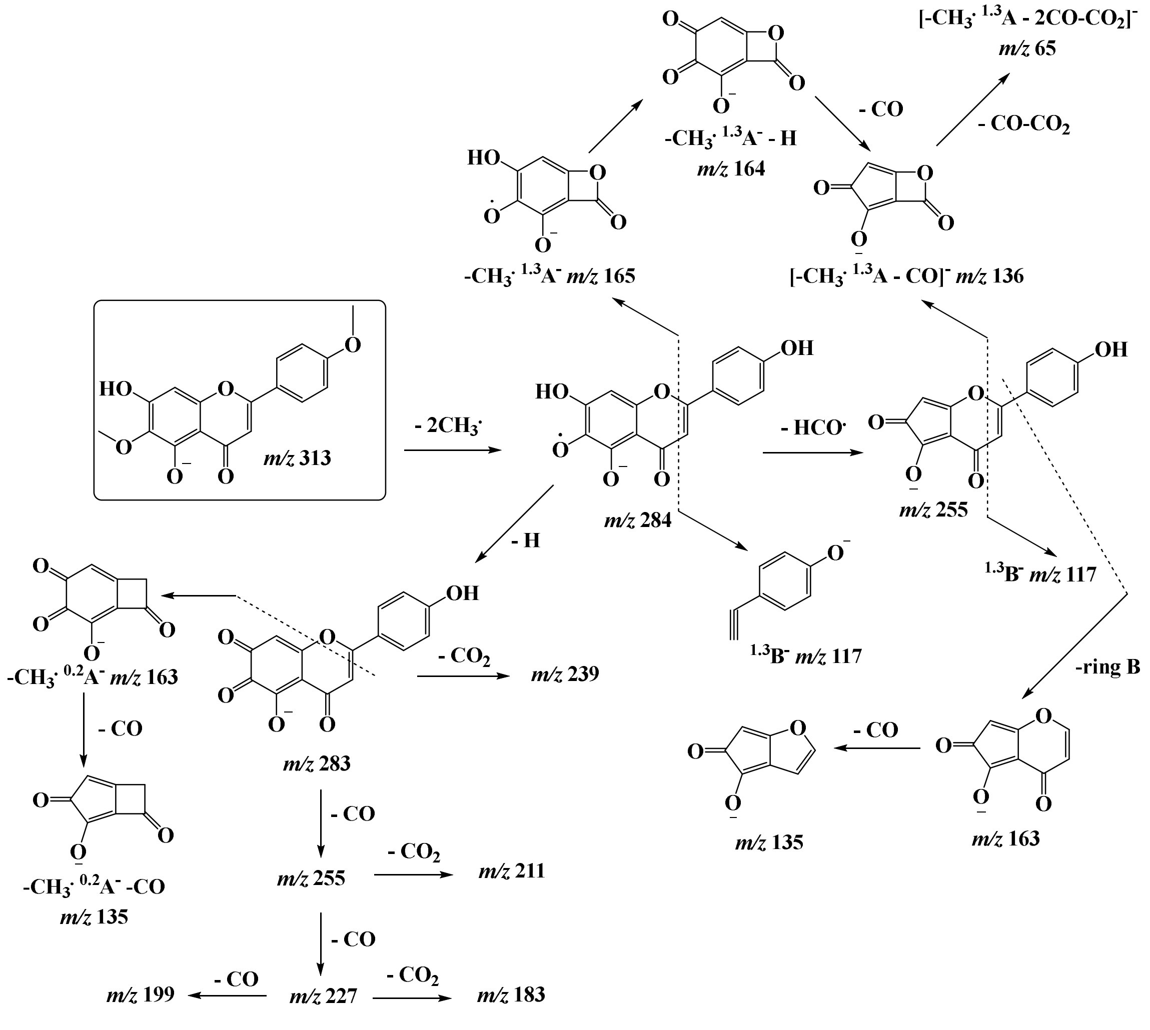

Figure S8. Proposed fragmentation of deprotonated pectolinarigenin [M-H]⁻ at CE 55 eV.

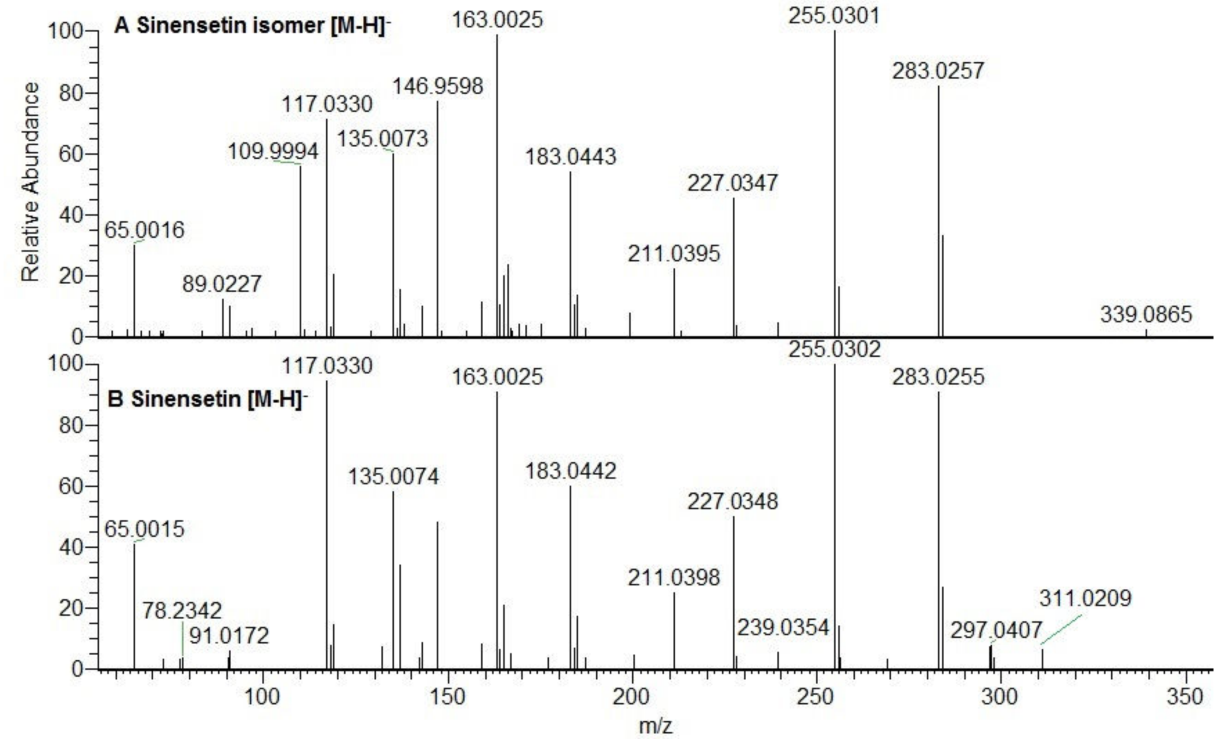

Figure S9. Mass spectrum of sinensetin and sinensetin isomer obtained by negative ion ESI-MS/MS.

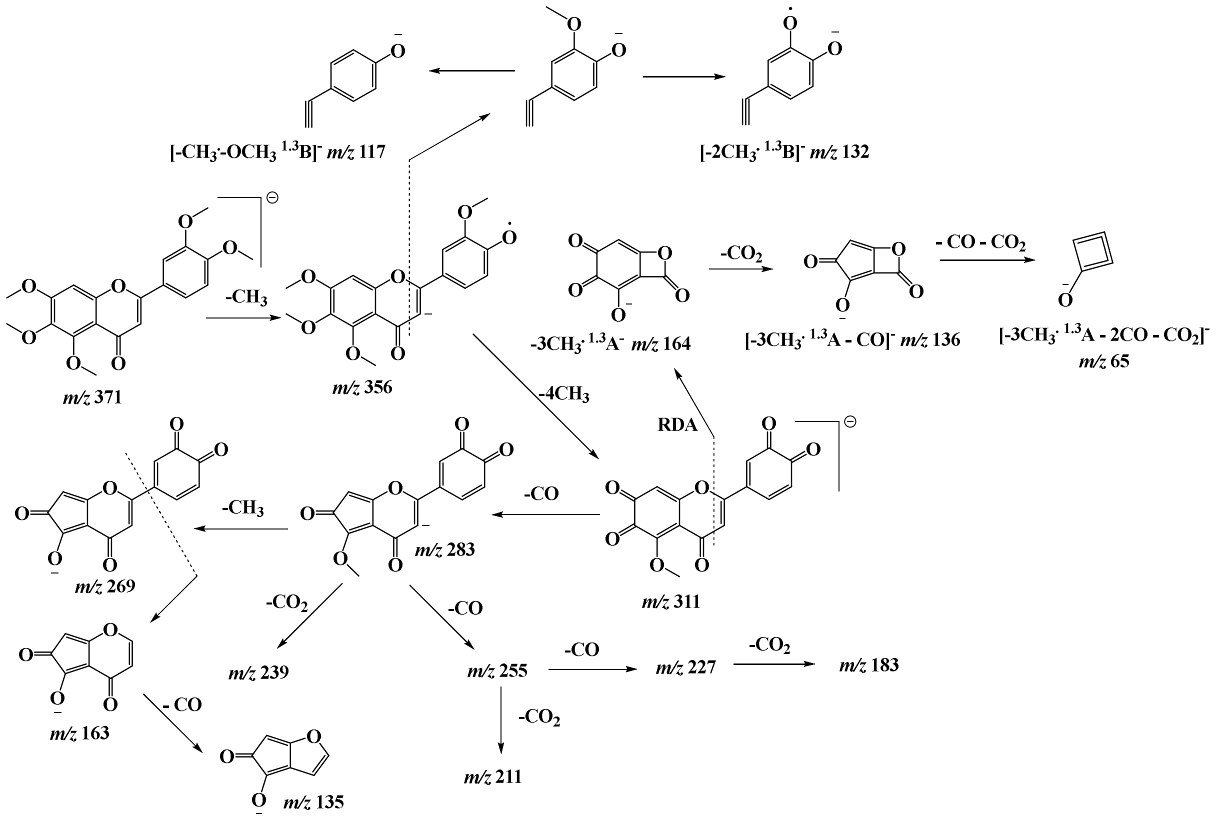

Figure S10. Proposed fragmentation of deprotonated sinensetin [M-H]$^-$ at CE 55 eV.

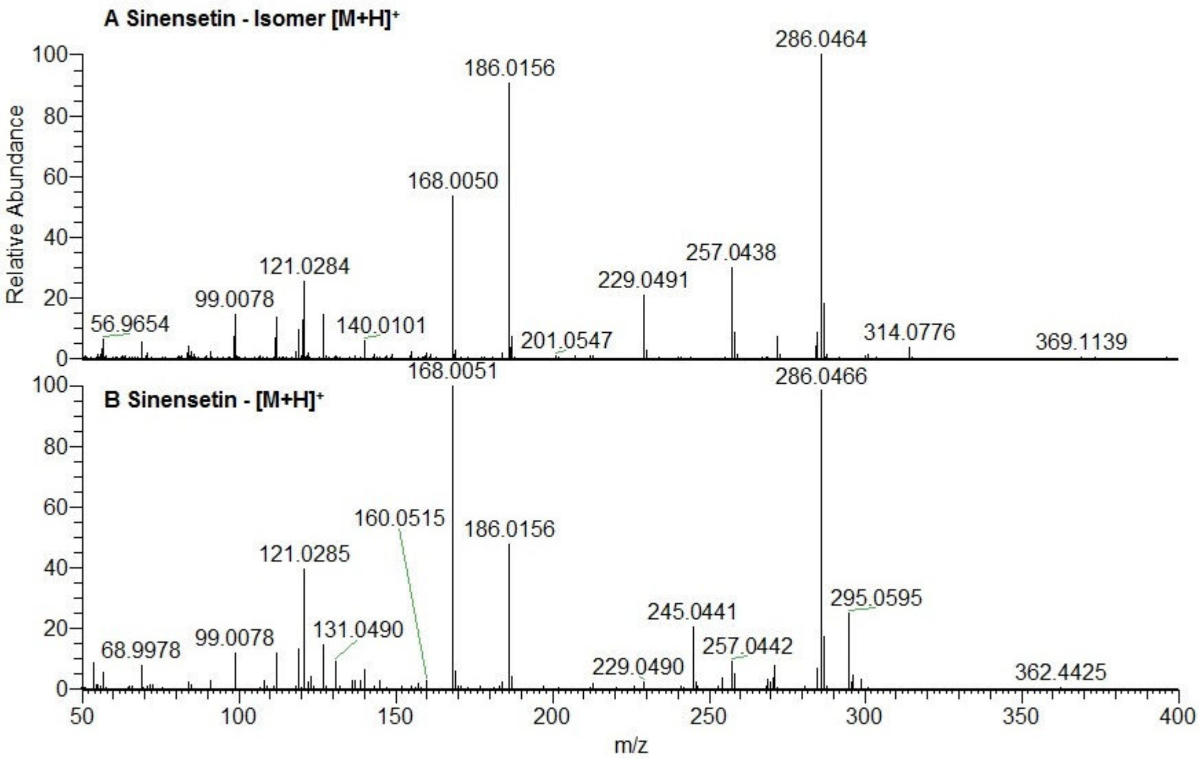

Figure S11. Mass spectrum of sinensetin and sinensetin isomer obtained by positive ion ESI-MS/MS.

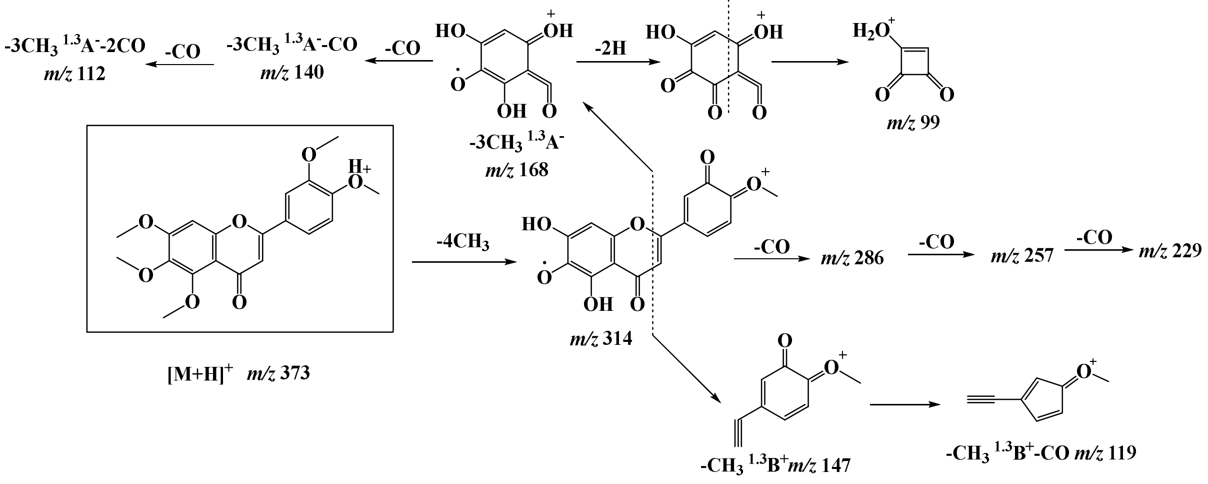

Figure S12. Proposed fragmentation of protonated sinensetin [M+H]+ at CE 60 eV.

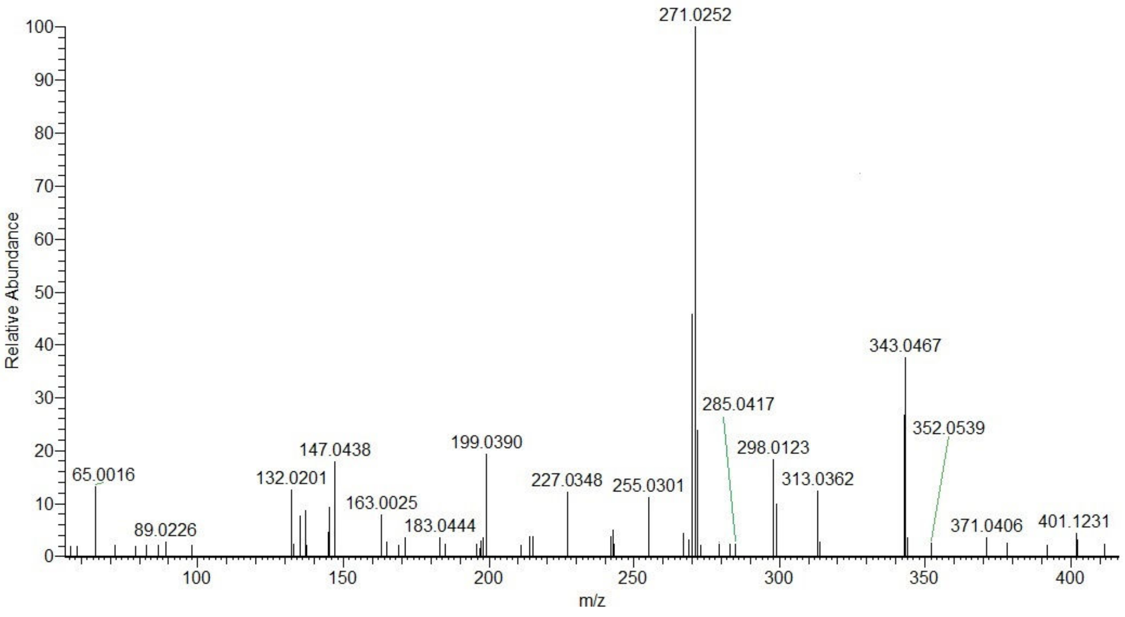

Figure S13. Mass spectrum of nobiletin obtained by negative ion ESI-MS/MS.

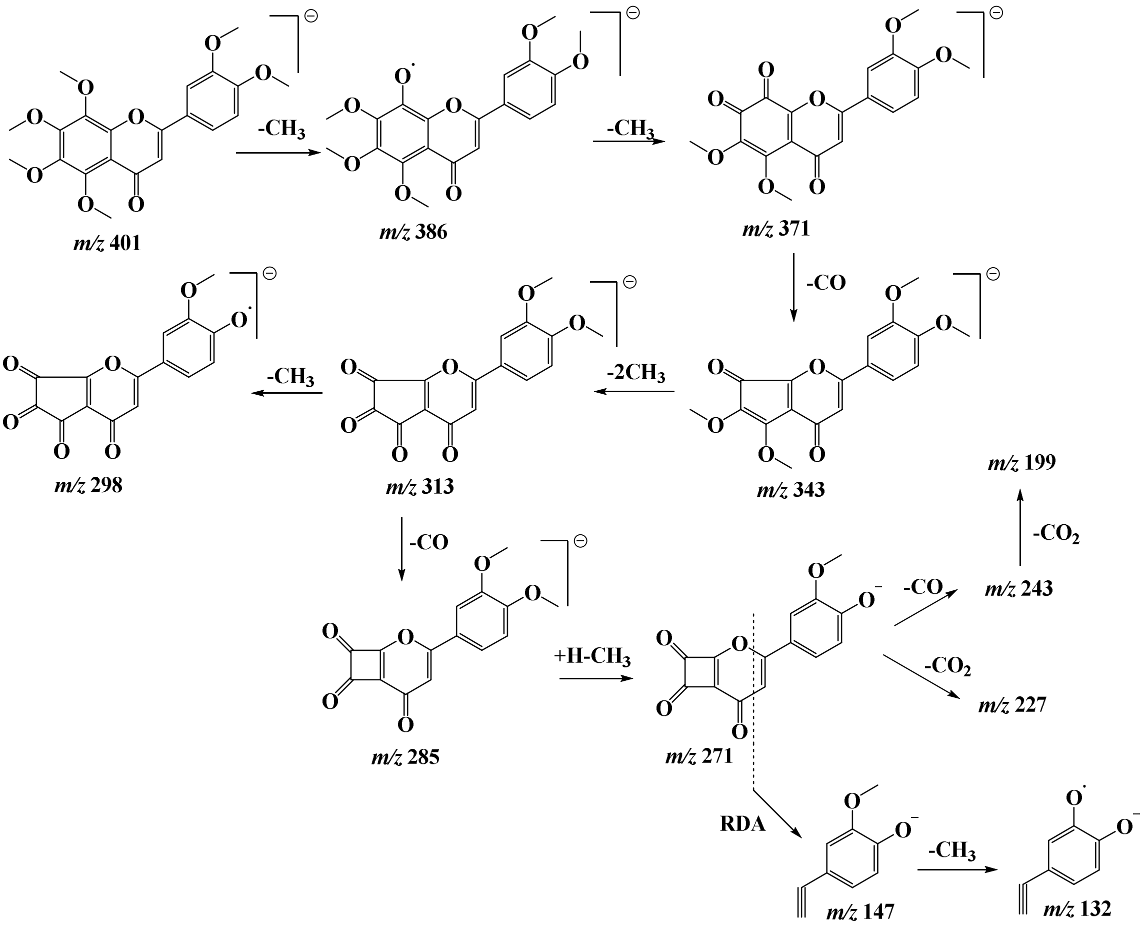

Figure S14. Proposed fragmentation of deprotonated nobiletin $[M-H]^-$ at CE 55 eV.

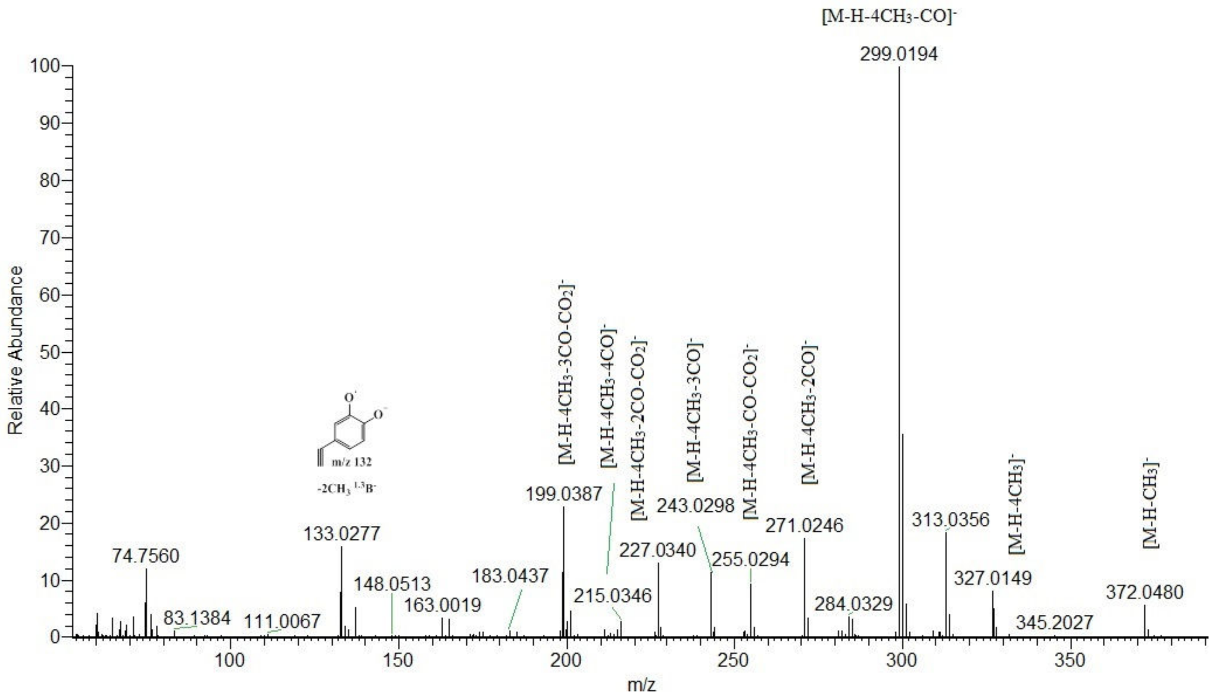

Figure S15. Mass spectrum of demethylnobiletin obtained by negative ion ESI-MS/MS.

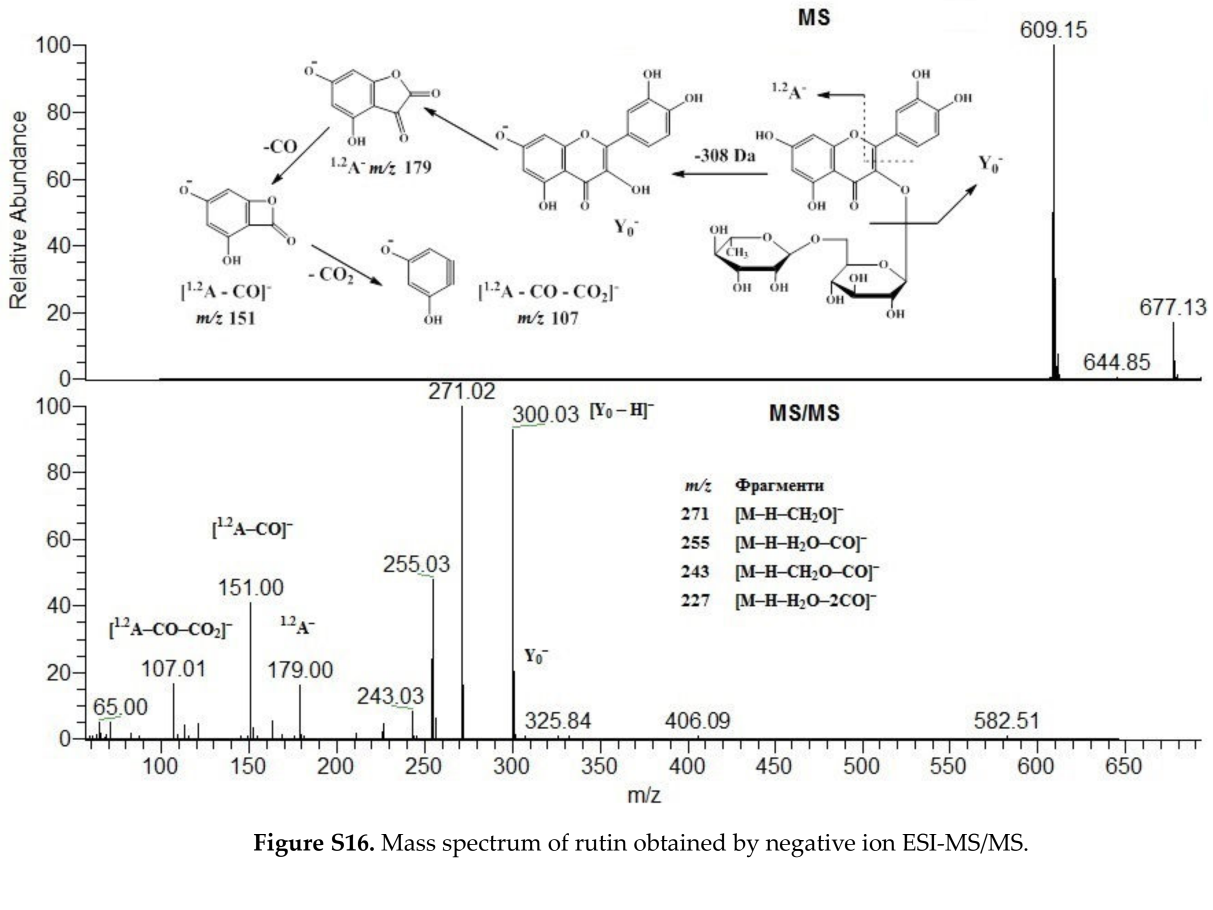

Figure S16. Mass spectrum of rutin obtained by negative ion ESI-MS/MS.

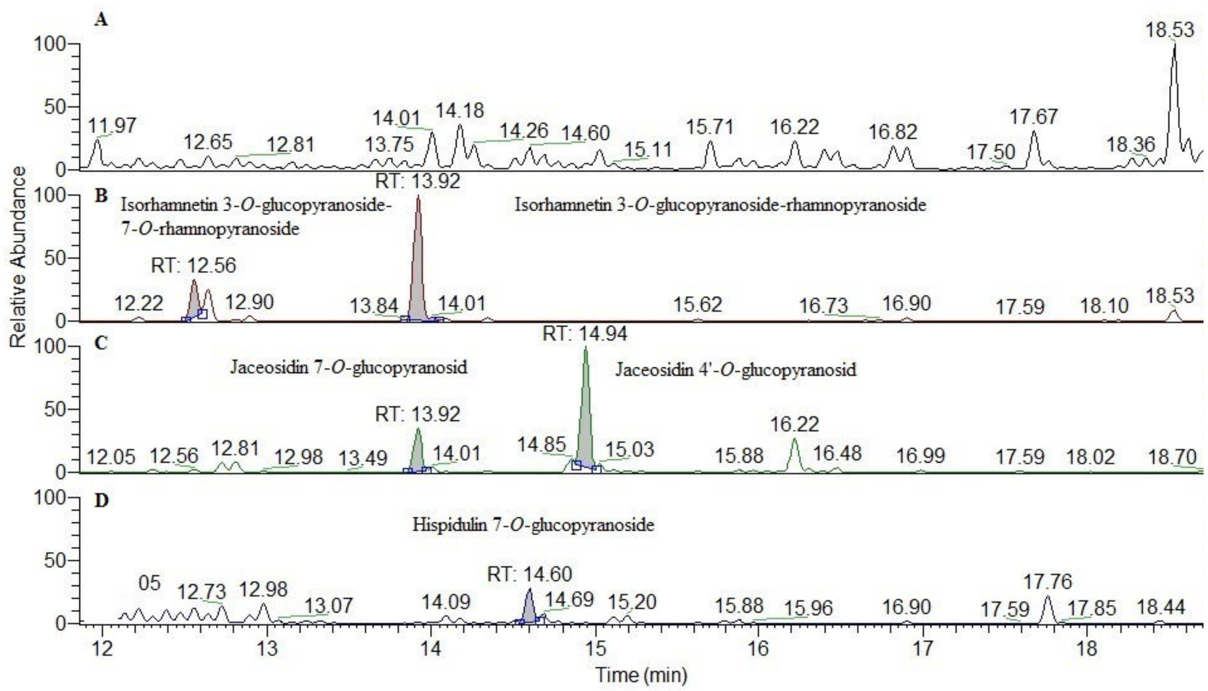

Figure S17. Total ion current (TIC) of flavonoids from *C. botrys* in EtOAc fraction (**A**); Profile of selected isorhamnetin glycosides (**B**); Profile of selected jaceosidin glycosides (**C**); Profile of selected hispidulin glycoside (**D**).
